# Large-scale real-world data on a multidisciplinary approach to spinal cord stimulation for persistent spinal pain syndromes: first evaluation of the Neuro-Pain^®^ nationwide screening and follow-up interactive register

**DOI:** 10.3389/fnins.2024.1322105

**Published:** 2024-03-22

**Authors:** Lisa Bernaerts, Ella Roelant, Frederic Lecomte, Maarten Moens, Jean-Pierre Van Buyten, Bart Billet, Bart Bryon, Martine Puylaert, Tuna Turgay, Maureen Malone, Tom Theys, Jan Van Zundert, Anne Berquin, Erwin Crombez, Olivier De Coster, Johan Vangeneugden, Huynh Giao Ly, Marleen Louagie, Guy Henri Hans

**Affiliations:** ^1^Multidisciplinary Pain Center, Antwerp University Hospital, Antwerp, Belgium; ^2^Clinical Trial Center (CTC), CRC Antwerp, Antwerp University Hospital, Antwerp, Belgium; ^3^National Institute for Health and Disability Insurance, Brussels, Belgium; ^4^Department of Neurosurgery, University Hospital Brussels, Brussels, Belgium; ^5^Multidisciplinary Pain Center, AZ Niklaas, Sint Niklaas, Belgium; ^6^Multidisciplinary Pain Center, AZ Delta, Roeselare, Belgium; ^7^Multidisciplinary Pain Center, AZ Turnhout, Turnhout, Belgium; ^8^Multidisciplinary Pain Center, Ziekenhuis Oost-Limburg, Genk, Belgium; ^9^Multidisciplinary Pain Center, Hôpital Erasme, ULB, Brussels, Belgium; ^10^Multidisciplinary Pain Center, AZ Klina, Brasschaat, Belgium; ^11^Department of Neurosurgery, University Hospitals Leuven, Leuven, Belgium; ^12^Department of Physical and Rehabilitation Medicine, Cliniques Universitaires UCL, St. Luc, Brussels, Belgium; ^13^Multidisciplinary Pain Center, Ghent University Hospital, Ghent, Belgium; ^14^Department of Neurosurgery, AZ Sint Maarten, Mechelen, Belgium

**Keywords:** spinal cord stimulation, neuropathic pain, long-term data, chronic pain, neurostimulator, nation-wide dynamic register

## Abstract

**Introduction:**

Spinal cord stimulation is a common treatment option for neuropathic pain conditions. Despite its extensive use and multiple technological evolutions, long term efficacy of spinal cord stimulation is debated. Most studies on spinal cord stimulation include a rather limited number of patients and/or follow-ups over a limited period. Therefore, there is an urgent need for real-world, long-term data.

**Methods:**

In 2018, the Belgian government initiated a nationwide secure platform for the follow-up of all new and existing spinal cord stimulation therapies. This is a unique approach used worldwide. Four years after the start of centralized recording, the first global extraction of data was performed.

**Results:**

Herein, we present the findings, detailing the different steps in the centralized procedure, as well as the observed patient and treatment characteristics. Furthermore, we identified dropouts during the screening process, the reasons behind discontinuation, and the evolution of key indicators during the trial period. In addition, we obtained the first insights into the evolution of the clinical impact of permanent implants on the overall functioning and quality of life of patients in the long-term.

**Discussion:**

Although these findings are the results of the first data extraction, some interesting conclusions can be drawn. The long-term outcomes of neuromodulation are complex and subject to many variables. Future data extraction will allow us to identify these confounding factors and the early predictors of success. In addition, we will propose further optimization of the current process.

## 1 Introduction

Spinal cord stimulation (SCS) is an invasive technique used to treat chronic neuropathic pain and other conditions. The first neurostimulator was implanted by Shealy et al. (1967), based on the Gate Control Theory of Melzack and Wall. Electrodes are placed along the dorsal aspect of the spinal cord in the epidural space and a small electronic pulse generator is implanted. Direct or facilitated inhibition of pain transmission or blocking of nerves is believed to be the underlying mechanism of pain control (Sdrulla et al., 2018; Jensen and Brownstone, 2019; Eisdorfer et al., 2020). SCS is a therapeutic option for patients with failed conservative care, including medication, infiltration, and rehabilitation, who have had spinal surgery but remain in pain. The use of a trial period is effective in determining the patients eligible for a permanent implantable device (Chincholkar et al., 2011). Reports show long-term efficacy in 37%–88% of implanted patients when effectiveness is defined as ≥50% pain relief (Caylor et al., 2019; Guzzi et al., 2022). Pain reduction, pain medication reduction, improved sleep quality, and increased activity levels are the generally expected outcomes. However, complications may occur during the trial period or after the permanent device implantation. Infection, lead migration, lead fracture, and scar tissue formation are the most frequent possible complications (Andersen, 1997; Spincemaille et al., 2000; Kleiber et al., 2016; Ratnayake et al., 2020; Pino et al., 2022). Nonetheless, SCS is a safe intervention, although careful patient selection and appropriate techniques are mandatory (Kleiber et al., 2016).

In addition to proper selection of patients, long-term follow-up remains challenging. Several studies have reported follow-up of up to 1 year or even longer after permanent implantation. O’Connell et al. (2021) reviewed 15 outcome-based studies (Andersen, 1997). The outcomes were evaluated at short-(≤1 month after permanent implantation), medium-(4–8 months), and long-terms (≥12 months). The authors found ≥50% pain relief in two short-term studies, five medium-term studies, and one long-term study. Kallewaard et al. (2021) evaluated 188 permanently implanted patients. Data were collected at the completion of the trial implantation and at 3 and 12 months after permanent implant implantation; 135, 117, and 90 patients, respectively, completed the follow-up. Approximately half of the implanted patients completed the final follow-up period. Significant pain reduction was observed at 3 and 12 months, and a substantial improvement in the quality of life (QoL) was observed at the last follow-up. A recent retrospective observational study investigated the long-term outcomes in 191 patients who received permanent SCS implant (Puylaert et al., 2023). At a mean follow-up of 10.6 years, 78.5% of the patients were satisfied with the treatment outcome. In a prospective study of 176 patients with chronic pain, repetitive evaluations were performed for up to 28 weeks after permanent implantation (Brill et al., 2022). Despite significant short-term improvements, the outcomes at the 7 months follow-up did not seem to differ from those of the control group (patients whose trial failed and did not receive any permanent implantation). Several authors have expressed concerns about possible biases in the study design; although the applied methodology was well conceived, the sample size was sufficiently large, and there was no industry support for conducting the study (Maarrawi, 2022; Hitt and deLeon-Casasola, 2023).

### 1.1 Pre-implantation psychosocial variables as predictors of SCS outcome

Several authors have highlighted the importance of pre-implantation psychosocial variables in SCS treatment. Psychological screening for “yellow flags” (indicating greater risk of progression of psychological distress and disability relating to pain and its management) is highly recommended and is becoming a common practice in the treatment of SCS. Celestin et al. performed a systematic review of 25 studies to examine the relationship between pre-surgical and pre-implantation variables and treatment outcomes in lumbar surgery and SCS (Celestin et al., 2009). In 92% of the studies, a positive relationship was found between one or more psychological factors and poor treatment outcomes. Pretreatment somatization, depression, anxiety, and poor coping were the most predictive factors for reduced benefits from surgical interventions or SCS. Pretreatment physical findings, activity interference, and pain intensity were minimally predictive. Bendinger et al. (2015) performed a retrospective analysis of 83 patients treated with SCS. Patients were divided into two groups: those with less than 50% pain reduction and those with more than 50% pain reduction at 1 year follow-up. Preimplantation sleep interference, depression, pain catastrophizing, and pain self-efficacy were significantly worse in the group with less than 50% pain reduction. Prabhala et al. (2019) developed the Psychological Evaluation Tool for Spinal Cord Stimulation Candidacy (PETSCSC) and examined 34 patients with a mean follow-up of 9.88 months. The tool included all psychological factors that the literature has shown to correlate with SCS outcomes. Significant improvements in pain and QoL related to pain, catastrophizing, and disability were observed at the latest follow-up visit. A significant correlation was found between the PETSCSC scores and SCS outcome measures. Molloy et al. (2006) investigated whether a combination of intensive cognitive-behavioral pain management and spinal implantable devices may have better outcomes. The findings indicated that the combination of both therapies was associated with significant improvements in affective distress, disability, self-efficacy, and catastrophizing, but not in pain intensity. These results strongly support the biopsychosocial perspective of chronic pain, in which different factors contribute to complex chronic pain problems and are differentially targeted by different modalities.

In addition to the value of psychological factors as essential criteria during the screening process, they are also valuable as outcome measures. The goal of SCS treatment should not be restricted to pain improvement but should also target better functional status, social and professional reintegration, and improved health related QoL. Doleys (2006) reported that SCS has become a highly specialized and effective therapeutic approach. Despite the advantages of the operational techniques and hardware technology, 20%–50% of patients with good results during the trial reported a loss of analgesia within 1 or 2 years after permanent implantation. This loss of the therapeutic effect occurred even if the device functioned perfectly from a technical standpoint. The authors suggested that psychological factors may have played an important role and highlighted the importance of understanding these psychological variables to improve SCS outcomes. However, the assessment and evaluation of these psychological factors are complex.

The magnitude of the psychological factors varies with the complexity of the disorder. Complex disorders tend to cause psychological distress, and the relationship between pain and psychological variables can be bidirectional (Zimmerman et al., 1996; Cotchett et al., 2015; Blackburn et al., 2019; Garcia et al., 2021). Psychological complaints can be secondary to pain and therefore resolve after treatment. Psychological assessment can, therefore, have several functions: identifying predictors of a successful outcome, assessing a patient’s psychosocial status, suggesting which factors can be ameliorated by therapy, and providing a baseline from which the improvement can be evaluated. Blackburn et al. (2016) further explored the correlation between presurgical psychological assessment and chronic pain reduction with SCS. The assessment of psychosocial variables is complex, and excluding all patients with depression, anxiety, somatization, and/or poor coping skills may also exclude potential patients who may largely benefit from SCS, since the chronic pain population shares these psychological characteristics. Such judgments should be made by interpreting the results of a formal psychological inventory against a rigorous evaluation of pain history, coping, thoughts, expectations, and treatment goals.

The aim of this publication is threefold. First, an overview will be provided of how a national interactive register can be introduced to screen, evaluate, and follow-up patients during their treatment with SCS in a multidisciplinary approach. The Neuro-Pain^®^ project is an example of how a collaboration among different medical specialists, psychologists, patients, and health insurers is facilitated through a centralized technology-enabled approach. Second, herein, we will discuss the first data analysis of the results of the 3 weeks trial period and long-term follow-up of patients suffering from persistent spinal pain syndromes. Third, the final scope will be the description of the relation between psychological factors, “yellow flags,” and outcome measures. The following hypotheses were investigated: “*Are yellow flags predictive of less recovery and less satisfaction after trial*” and “*Are yellow flags predictive of lower functioning after 6 months and 1.5 years*.”

## 2 Materials and methods

### 2.1 Development and implementation of the Neuro-Pain^®^ platform

**Figure 1:**
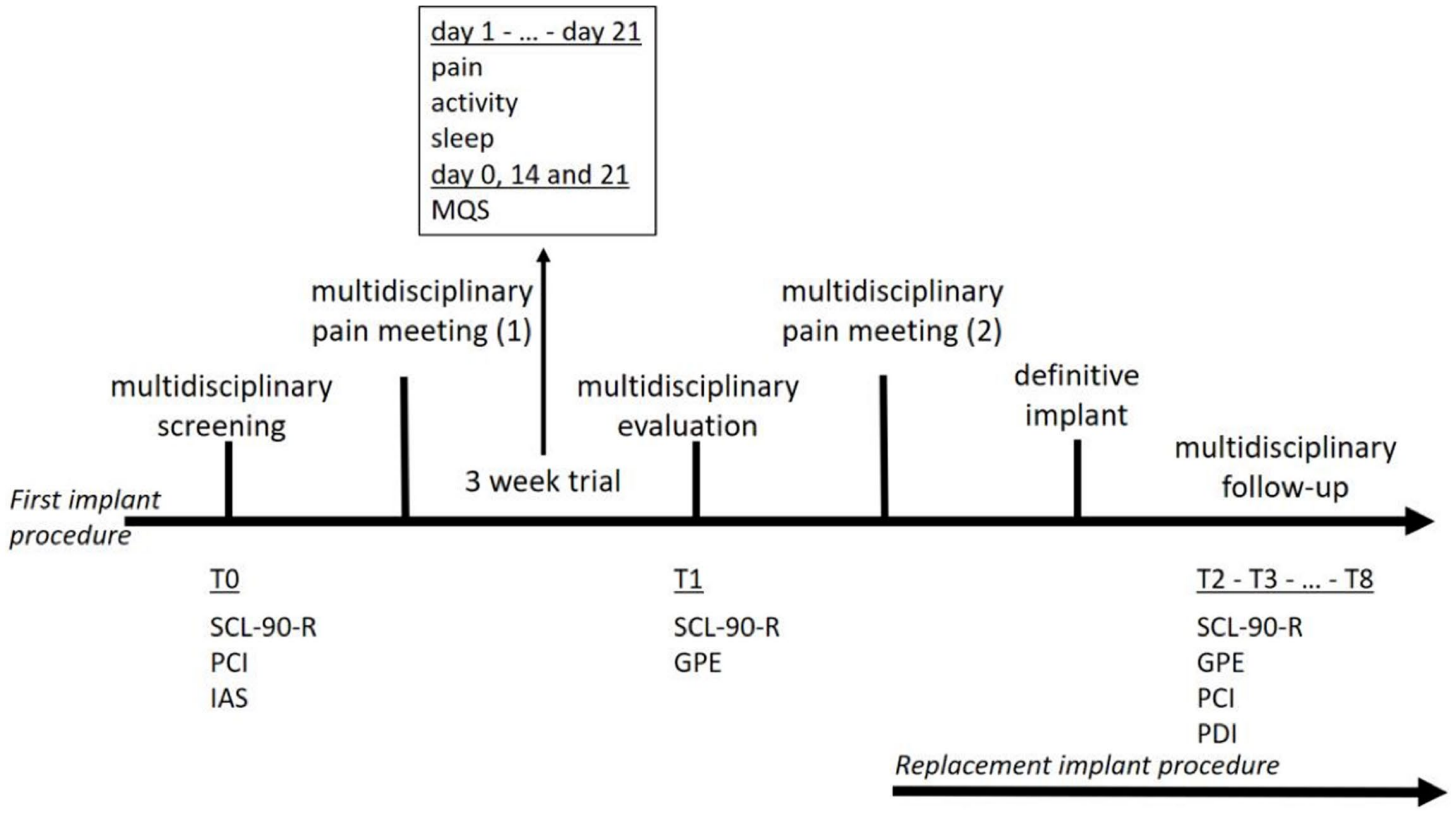
Course of treatment for a first implant procedure (first line) and a replacement implant procedure (second line).

In 2016, the Belgian government decided that a reorganization of the reimbursement procedure for SCS was necessary because of concerns regarding the growing financial impact and following a report by the Belgian Health Care Knowledge Center (KCE Report 189C). Together with the Belgian Pain Society (BPS) a task force was installed to redesign the screening and follow-up processes. After extensive multidisciplinary consultations, the national health authorities approved the revised procedure. The revised process was unique in terms of two elements: a multidisciplinary approach was embedded throughout the process and the entire procedure was concentrated in a centralized interactive nationwide register. A Royal Decree was published to implement this new procedure, which was initiated on February 16, 2018. The indications for new implants were limited to failed back and neck surgery syndromes (now referred to as persistent spinal pain syndrome type 2—PSPS type 2). The first step in the revised procedure was completion of the clinical file. Following this crucial step, a multidisciplinary evaluation was initiated (Figure 1). Subsequently, each patient case was discussed during a broad multidisciplinary pain meeting (MAO); the decision to stop or initiate a trial period was made by the entire care team. The new Royal Decree prescribed a minimum duration of 21 days for the trial period instead of a minimum 4 weeks trial period that was previously legally required. During the trial, patients were required to complete a diary of pain intensity, sleep quality, and level of physical activity. After 2 and 3 weeks of trial period initiation, the analgesic medication intake was evaluated. After the trial period, a new multidisciplinary evaluation was performed. Subsequently, a second MAO was convened, wherein the decision was made to either stop the procedure or proceed to permanent SCS implantation.

### 2.2 Recruitment of patients

All patients were treated at Belgian pain centers. Since January 2018, all patients who started treatment with SCS and those who needed battery replacement were included in the Neuro-Pain^®^ platform and provided informed consent to use their data for evaluating the technique. Currently, there are 35 recognized multidisciplinary pain centers in Belgium. All non-recognized multidisciplinary pain centers must cooperate with recognized multidisciplinary pain centers to include patients in SCS programs.

### 2.3 First implant procedure

Patients eligible for SCS were referred for multidisciplinary screening (T0). The pain specialists and neurosurgeons judge whether a patient is a good candidate for SCS. The patients were then referred for psychological pre-evaluative screening, which consisted of two consultations with a pain psychologist and a set of questionnaires measuring general psychological functioning, pain coping, and illness anxiety (R Core Team, 2021). Psychiatric advice was requested only when psychiatric illnesses (e.g., clinical depression or addiction) were present. Patients were invited to register to a web-based platform, Neuro-Pain^®^, where they completed the questionnaires. When all the information was assembled, a Multidisciplinary Pain Meeting was held with a pain specialist, neurosurgeon, and pain psychologist (psychiatrist). A decision was made on whether the patient met the medical and psychological criteria for SCS and whether a multidisciplinary consent was provided to start the trial period. When approved, the patients completed a 3 weeks trial period with implanted electrodes and an external battery. The patients were invited to the Neuro-Pain^®^ platform to complete a diary on their pain and activity levels, and sleep quality. Medication use was assessed on days 14 and 21. The attending physician documented the patient’s medication use on the implantation date (day 0). Medical and psychological evaluations were conducted after the trial (T1). Patients were invited to the Neuro-Pain^®^ platform to complete questionnaires on their psychological functioning, sense of recovery, and satisfaction with the treatment. During a second Multidisciplinary Pain Meeting (MAO) the patients’ results during the trial period were discussed. A successful trial period included reductions in pain and pain medication use, improved sleep quality, and increased activity levels. The implantation of an Internal Pulse Generator (IPG) was performed after obtaining multidisciplinary consent. After definitive implantation, the patient was asked—by means of an automated messaging system—to complete the questionnaires every 6 months (T2–T8). Although evaluating the patient’s global functioning with the implant was highly recommended, this online multidisciplinary follow-up was not mandatory. Patients do agree to a mandatory 2 years follow-up with their treating team at the start of the neurostimulation program. If however necessary, this is done more frequently.

### 2.4 Replacement implant procedure

Patients who underwent SCS treatment but required battery replacement were also included in the Neuro-Pain^®^ platform. The new IPG was implanted after obtaining consent from the multidisciplinary team. The patient was also invited to complete the questionnaires every 6 months (T2–T8). This follow-up was not mandatory but was highly recommended to evaluate the patient’s further functioning with the permanent implant. The questionnaires were identical to those used during the first implant procedure.

### 2.5 Web-based secured interactive register

**Figure 2:**
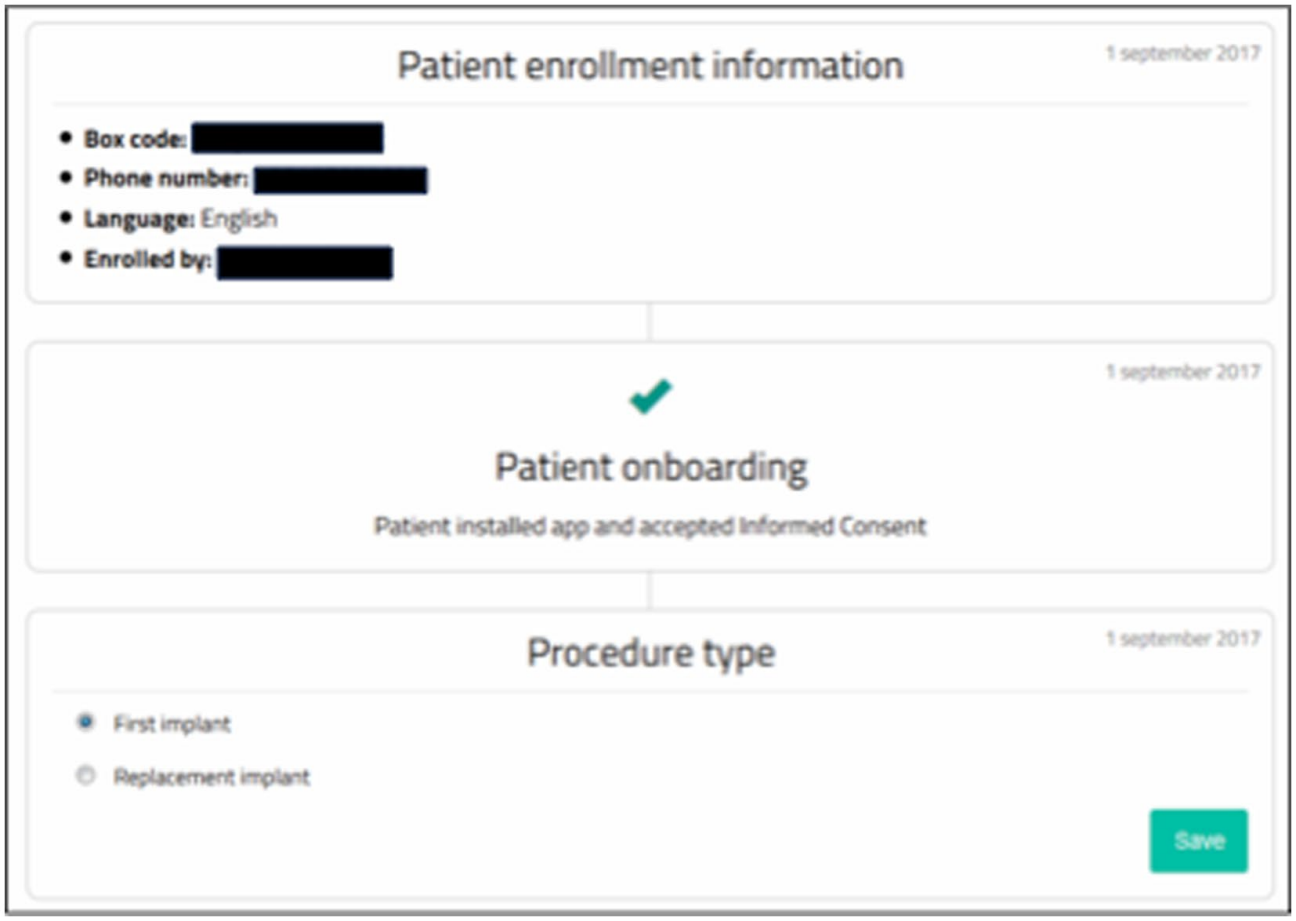
Neuro-Pain online platform, view from health care provider.

The Neuro-Pain^®^ online platform (Figure 2) was developed by the National Institute for Health and Disability Insurance (NIHDI) and the Belgian Pain Society (BPS). The technical development of the platform was performed by BeWell Innovations^®^ (Ranst, Belgium). The platform is hosted on the Antwerp University Hospital (UZA) servers. Neuro-Pain^®^ allows for collaboration between parties through an innovative web-based platform. Medical specialists, psychologists, patients, and health insurers have online access to the patient files. Patients become active participants in their treatment and are encouraged to track and adjust their own evolution. The requests for reimbursement are structured, and all patients undergo the same screening, trial, and follow-up modules.

### 2.6 Screening measures

A pre-evaluative psychological inventory was developed using the Symptom Checklist-90-Revised (SCL-90-R), Pain Coping Inventory (PCI), and Illness Attitude Scale (IAS). Each patient underwent two psychological pre-evaluative screening consultations conducted by a pain psychologist. During the 3 weeks trial period, patients completed a daily survey on pain, activity, and sleep. The attending physician completed the patient’s Medication Quantification Scale (MQS-IIIR) on day 0, and patients updated their medication status on days 14 and 21. After the trial was completed, a post-evaluative psychological inventory was created using the SCL-90-R and General Perceived Effect (GPE). All patients underwent one psychological post-evaluation consultation. Pre- and post-evaluations were mandatory for all patients as stipulated in the Royal Decree. Thereafter, although not mandatory, the patients were asked to complete the chronic follow-up questionnaires every 6 months after a definitive implantation. This chronic follow-up was performed using the SCL-90-R, GPE, PCI, and Pain Disability Index (PDI). The questionnaires were completed using a patient-specific online platform. The platform and corresponding questionnaires are available in the three national languages—Dutch, French, and German—and the patients could choose their preferred language.

#### 2.6.1 Symptom Checklist-90-Revised

The SCL-90-R is a multidimensional inventory of physical and mental health complaints, developed by Derogatis et al. (1973). The Dutch items are rated on a 5-point Likert scale ranging from 1 (not at all) to 5 (extremely), specifying the extent to which each complaint has bothered the patient during the past 7 days. The French and German items are rated on an analogue 5-point Likert scale ranging from 0 (not at all) to 4 (extremely) (Fortin et al., 1989; Schmitz et al., 2000). The SCL-90-R consists of 90 items. The Dutch version has one total score and eight subscales: agoraphobia, anxiety, depression, somatic complaints, thinking insufficiency, interpersonal sensitivity, hostility, sleep problems, and additional items. Psychoneuroticism is a total score that reflects the general physical and psychological dysfunctioning (Pedersen et al., 2016). The French and German versions represent three total scores and nine subscales: somatization, obsessive-compulsiveness, interpersonal sensitivity, depression, anxiety, hostility, phobic anxiety, paranoid ideation, and psychoticism. The Global Severity Index, Intensity of Reported Symptoms, and Number of Reported Symptoms comprise the three total scores. The Global Severity Index is considered the most sensitive single quantitative indicator of a patient’s psychological distress status. The questionnaire covers an essential part of the complaints that can be found in an outpatient psychiatric population and is a convenient screening and evaluation tool for treatment outcome (Geiser et al., 2000; Schmitz et al., 2000; Kostaras et al., 2020). Standardized norms are available for the Dutch version of the Chronic Pain Population.

#### 2.6.2 Pain Coping Inventory

The PCI consists of six scales measuring cognitive and behavioral pain-coping strategies representing two higher-order pain-coping dimensions: active and passive coping (Jensen et al., 1995; Hadjistavropoulos et al., 1999; Kraaimaat and Evers, 2003). The questionnaire comprises 33 items. The items are rated on a 4-point Likert scale ranging from 1 (hardly ever) to 4 (very often). Patients were asked how frequently they used a specific coping strategy when dealing with pain. Active pain-coping strategies reflect three cognitive-behavioral strategies: measuring patients’ efforts to distract themselves from pain (distraction, five items), to reinterpret and transform pain (pain transformation, four items), and to function despite pain (reducing demands, three items). Passive pain coping reflects three cognitive-behavioral strategies: behavioral tendencies to restrict functioning (resting, five items), avoiding environmental stimuli (retreating, seven items), and catastrophic cognitions about pain (worrying, nine items). A composite score of the active and passive coping dimensions is calculated by summing the non-weighted scores of the three active and passive coping strategies. We used the Dutch, French, and German translations of the original version (Truchon et al., 2006; Hechler et al., 2008).

#### 2.6.3 Illness Attitude Scale

The Illness Attitude Scale (IAS), originally developed by Kellner (1987), measures the fears, attitudes, and beliefs associated with hypochondriacal concerns and abnormal illness behavior. The Dutch, French, and German versions of the IAS were used (Speckens et al., 1996a,b). The questionnaire comprises 29 items. The items are rated on a 5-point Likert scale ranging from 0 (no) to 4 (generally). The Dutch population showed two underlying factors, leading to two subscales: illness anxiety and illness behavior. For the Dutch version, standardized norms are available for the general population and for the general practitioner population. These norms are currently missing for the French and German versions.

#### 2.6.4 Diaries

The patients completed three daily diary questions during the trial period: pain, activity, and sleep. The patients indicated pain by sliding a marker point along a line. The endpoints were defined as “no pain” and “the worst imaginable pain”; higher scores indicating greater pain. They indicated their activity levels by sliding a marker point on a line. The endpoints were defined as “not active” and “very active”; higher scores reflecting higher activity levels. Finally, the sleep quality was indicated by sliding a marker point on a line. The endpoints were defined as “bad” and “excellent”; higher scores indicated better sleep quality. The diary questions were completed daily.

#### 2.6.5 Medication Quantification Scale

The III-R version of the MQS medication quantification scale was used to quantify medication regimen use in patients with chronic pain (Harden et al., 2005; Gallizzi et al., 2008). Previously, the MQS-III was used to correlate the amount and type of pain medications (analgesic drugs and all possible adjuvant drugs) prescribed to patients with complex regional pain syndromes (CRPS) and low back pain (Gallizzi et al., 2015; Goudman et al., 2020). In our setting, the MQS-III was used to measure possible reductions in medication use as an indicator of treatment success.

#### 2.6.6 Global Perceived Effect

The Global Perceived Effect (GPE) is a short questionnaire that measures the degree of recovery and patient satisfaction after medical treatment (Hudak and Wright, 2000), and consists of two items. The first item asks about the extent to which the patients feel recovered from their symptoms since the beginning of treatment. The answers are given on a 7-point numeric scale, ranging from “very much improved” to “very much deterioration.” The second item assesses the extent to which the patients feel satisfied with the treatment. The answers are given on a 7-point numeric scale, ranging from “absolutely satisfied” to “absolutely dissatisfied.” Original Dutch, French, and German translations were used (Hudak and Wright, 2000).

#### 2.6.7 Pain Disability Index

The PDI is a brief questionnaire measuring disability or the impact of pain symptoms on a person’s life (Pollard, 1984). It measures the effect of pain on a person’s ability to participate in essential life activities. The questionnaire consists of seven items, each representing one subfield: family/home responsibilities, recreation, social activities, occupation, sexual activities, self-care, and life support activities. The answers are given on an 11-point numeric scale, ranging from “no limitations” to “totally limited.” The scores for each item are summed to obtain the total score; higher scores indicate more limitations due to pain. We used authorized Dutch, French, and German translations (Dillmann et al., 1994; Gauthier et al., 2008; Langenfeld et al., 2022; van der Gucht et al., 2022).

### 2.7 Statistical analysis

Data collection using the Neuro-Pain^®^ platform began on February 14, 2018. Data extraction, on which this report was based, took place on July 14, 2022, and data cleaning was not performed. For 330 records, no further information was available (in addition to the initial demographic data); therefore, these records were excluded from all further analyses.

A total of 7,304 records from 6,170 different patients were included in the study. A single patient can have multiple records (e.g., one first implant procedure and two consecutive replacements). The date of birth was entered into the database. The age (at the time of data extraction) and sex were reported. Nineteen patients were excluded from the calculation of the mean and standard deviation (SD) of age because of a reported age <7 years. The properties of the 7,304 records are described in this section.

There were in total 3,191 patients for whom a procedure for the first implant was activated. There were 2,601 records with pre-evaluation, trial, and post-evaluation, describing 2,572 different patients. In case of duplicates, the most complete record was considered for the patient. For nine duplicates, the completion rate was equal across the two records, and the first entry was considered. This dataset of 2,572 patients was used for further analysis and was labeled as *Sub dataset* in the following analyses.

The number of patients who completed the follow-up questionnaires every 6 months after the definitive device implantation varied. The data on the 6 months follow-up measurements after permanent implant were analyzed as recorded in the system using the consecutive number of follow-ups as the indicator for time points filled in. The number of completed questionnaires and the procedure in which the patient was involved (first or replacement implant procedure) were reported.

Two free-text boxes (replacement for another diagnosis and preterm ending of the trial period) were quantified and analyzed. These data points were represented in a tabular format, with each row representing the procedure for a particular patient. The clinical diagnosis of pain was entered as a free text, and symptom categories [neuropathy, algoneurodystrophy (complex regional pain syndrome), post-trauma, polyneuropathy, postoperative, sciatica, headache, backache, facial palsy, and pancreatitis] were retrieved. If the reason for stopping the procedure was “infection,” this could be retrieved in another free-text field. These categories were represented in binary format, indicating the presence or absence of symptoms. The detection method was a dictionary-based approach in which the source data were iteratively scanned to find a list of triggers for each symptom. This approach ensured the detection of linguistic variations, negation terms, and languages used within the free-text field between patients. The symptoms were evaluated manually while compiling a programmable Python script. This process ensured the repeatability of an extended dataset.

The group of patients who stopped the procedure after the first Multidisciplinary Pain Meeting was compared with those who continued using an unpaired *t*-test (with equal or unequal variances) or the Mann–Whitney test, as appropriate. The *p*-values were corrected for multiple testing using the Bonferroni–Holm correction, which was only done for the three subscales of the PCI Active Coping, three subscales of the PCI Passive Coping, eight subscales of the Dutch version of the SCL-90-R, nine subscales of the French and German versions of the SCL-90-R, and two subscales of the IAS.

The patients completed three daily diary questions during the trial period: pain, activity, and sleep. The values were summarized per week, and these three measurements were then modeled using a linear mixed model, with time as a fixed effect and the subject as a random effect. In the case of a significant time effect, pairwise post hoc comparisons of the three time points were considered using Tukey’s correction for multiple testing. The same model was used for the MQS measurements on days 0, 14, and 21. The other outcomes (SCL-90-R, GPE, PDI, and PCI) were also measured multiple times and analyzed in a similar way using a linear mixed model with time as a fixed effect and subject as a random effect using all the available measurements. In the case of a significant time effect, post hoc tests were performed, where consecutive time points were compared. A Bonferroni–Holm correction was used for the *p*-values of the time effect for the subscales and post hoc comparisons.

To investigate the hypothesis that yellow flags were predictive of less recovery and satisfaction after the trial period, a logistic regression model was fit with recovery and satisfaction as binary outcomes (above 4 is outcome 1 and 4 or less is outcome 0) and yellow flags as predictors. Bonferroni–Holm multiple testing correction was performed on the subscales, as previously described. A similar logistic regression model was used to investigate whether yellow flags predicted lower functioning after 6 months and 1.5 years. For this purpose, disability was categorized into two categories: above (outcome 1) or below (outcome 0) the median PDI (51 in this case). Alternative statistical analyses were performed for sensitivity analysis. An ordinal regression model was fit with recovery and satisfaction as ordinal outcome measures (1–7) and the yellow flags as predictors, and a linear regression model with disability as a continuous outcome measure (0–100) was considered to investigate if the yellow flags were predictive of lower functioning after 6 months and 1.5 years.

Statistical significance was set at *p* < 0.05. Significant *p*-values are shown in bold, and the analyses were performed using R 4.1.2 (Federale overheidsdienst sociale zekerheid, 2017).

## 3 Results

### 3.1 Participants

The mean age of the included patients was 57.7 years with an SD of 11.4 years, the minimum and maximum ages were 20 and 93 years, respectively. There were 3,811 females (61.8%), 2,358 males (38.2%), and one other (0.02%). Fifty-five and six tenths percent of records were related to the replacement procedure and 44.4% to the primo-implant implant procedure. In the primary implant procedure, 90.3% of records had a diagnosis of Failed Back Surgery Syndrome (FBSS), and 9.7% had a diagnosis of Failed Neck Surgery Syndrome (FNSS). Among the replacement procedures, 80.2% were diagnosed with FBSS, 7.7% were diagnosed with FNSS, 0.7% were diagnosed with Complex Regional Pain Syndrome (CRPS), and 11.4% had another diagnosis. The last group of 461 records was further examined by screening the textbox, in which the physician could enter other diagnoses for several common indications for SCS. The results showed that in 266 records patients were treated for “neuropathy,” 35 were treated for “algoneurodystrophy (CRPS),” 32 for “post-trauma,” 6 for “polyneuropathy,” 29 for “postoperative,” 24 for “sciatica,” 12 for “headache,” 2 for “back pain,” 1 for “pancreatitis” and 2 for “facialgia.” Patients’ language (Dutch, French, or German) was determined based on the language chosen during the completion of the first questionnaire. Of the patients, 74.1% chose Dutch, 25.6% chose French, and 0.3% chose German to complete the questionnaire. These percentages were based on the patients who completed the questionnaires; however, 2,445 patients did not complete any questionnaires.

Regarding follow-up questionnaires to be completed every 6 months after the definitive device implantation, based on the first entry of each patient, 2,801 patients (45.4%) did never complete the questionnaire. Three thousand three hundred sixty-nine patients (54.6%) completed at least once the questionnaire of the chronic follow-up. Table 1 shows the number of completed questionnaires during the different follow-up periods.

**Table 1:** Number of patients completing the chronic follow-up questionnaires (by procedure).

Number of follow-ups	Number of patients (%)
0	2,801 (45.4%)
1	3,369 (54.6%)
2	1,749 (28.3%)
3	1,096 (17.8%)
4	744 (12.1%)
5	412 (6.7%)
6	205 (3.3%)
7	55 (0.9%)

### 3.2 Multidisciplinary Pain Meeting (1) (T0)

After the screening was completed, the Multidisciplinary Pain Meeting approved 99% of the records for trial implants. The procedure was stopped in 1% of the screened patients. After multiple testing corrections, only the total score of the Dutch version of the SCL-90 Psychoneuroticism differed significantly between patients who stopped before the initiation of the trial and those who continued the trial period (see Table 2). Interpreting the Dutch scores by applying the norms of the chronic pain population, the total score of the SCL90 Psychoneuroticism of those who stopped before initiating the trial period was clinically higher than that of those who continued the procedure (see Table 3).

**Table 2:** Comparison of psychological pre-evaluative screening measures for stop group and continue group (T0).

Psychological pre-evaluative screening	*n* stop group	Mean (T0)	SD	*n* continue group	Mean (T0)	SD	Raw *p*-value *t*-test (°)	Corrected *p*-value
SCL90 agoraphobia^a^	29	10.1	15.6	1,873	10.0	15.3	0.981	0.981
SCL90 anxiety^a^	29	23.1	19.3	1,873	18.3	15.5	0.103	0.620
SCL90 depression^a^	29	36.4	24.0	1,873	25.8	18.7	**0.025**	0.202
SCL90 somatic complaints^a^	29	43.6	13.0	1,873	37.4	15.1	**0.029**	0.202
SCL90 insufficiency^a^	29	40.2	18.8	1,873	35.6	18.3	0.182	0.814
SCL90 sensitivity^a^	29	17.2	19.2	1,873	13.2	14.1	0.265	0.814
SCL90 hostility^a^	29	13.9	14.1	1,873	11.5	12.9	0.319	0.814
SCL90 sleep problems^a^	29	68.4	26.5	1,873	61.1	27.9	0.163	0.814
SCL90 Psychoneuroticism^a^	29	27.7	15.8	1,873	22.6	13.1	**0.038**	**0.038**
PCI total active coping	31	44.2	14.1	2,569	44.6	14.7	0.875	0.875
PCI pain transforming	31	37.6	24.2	2,569	38.6	21.8	0.804	1.000
PCI distraction	31	46.7	14.7	2,569	48.6	18.7	0.570	1.000
PCI reducing demands	31	49.1	25.3	2,569	46.1	26.4	0.527	1.000
PCI total passive coping	31	51.1	13.3	2,569	49.1	16.3	0.507	0.507
PCI retreating	31	37.8	17.8	2,569	37.1	20.3	0.843	1.000
PCI worrying	31	53.3	19.0	2,569	49.9	20.1	0.361	1.000
PCI resting	31	65.6	18.4	2,569	64.4	22.0	0.774	1.000
IAS illness anxiety	31	12.5	7.9	2,569	10.0	7.9	0.077	0.154
IAS illness behavior	31	17.2	2.9	2,569	16.7	3.5	0.444	0.444

For SCL90 depression and SCL90 sensitivity, Welch’s test was used (unequal variance).

a Dutch version and scoring of the SCL-90-R. Values in bold indicate statistically significant differences.

**Table 3:** Comparison of psychological pre-evaluative screening measures for stop group and continue group (T0).

Psychological pre-evaluative screening	Interpretation stop group	Interpretation continue group
SCL90 agoraphobia^a^	Average	Average
SCL90 anxiety^a^	High	Average
SCL90 depression^a^	High	Above average
SCL90 somatic complaints^a^	High	Above average
SCL90 insufficiency^a^	High	Above average
SCL90 sensitivity^a^	High	Above average
SCL90 hostility^a^	Average	Average
SCL90 sleep problems^a^	High	Above average
SCL90 Psychoneuroticism^a^	High	Above average

Interpretation norms of the chronic pain population.

a Dutch version and scoring of the SCL-90-R.

### 3.3 Outcome measures during and after the 3 weeks trial period

Analyzing the Sub dataset, the evolution of the weekly measures (mean ± SD) during the trial period of the three variables included in the diaries, pain, sleep, and activity, are provided in Table 4. The mean (SD) scores on the MQS-IIIR on days 0, 14, and 21 were 19.19 (9.93), 12.72 (8.31), and 11.88 (7.77), respectively, indicating a decrease in the intake of the pain medication during the trial period.

**Table 4:** Means (SD) of weeks 1, 2, and 3 for the three diary variables pain, sleep, and activity.

Diary	Mean week 1 (SD)	Mean week 2 (SD)	Mean week 3 (SD)
Pain	4.69 (2.08)	3.61 (2.00)	3.05 (1.95)
Sleep	4.91 (2.02)	6.1 (2.09)	6.58 (2.13)
Activity	3.57 (1.75)	4.99 (1.89)	5.62 (2.01)

**Figure 3:**
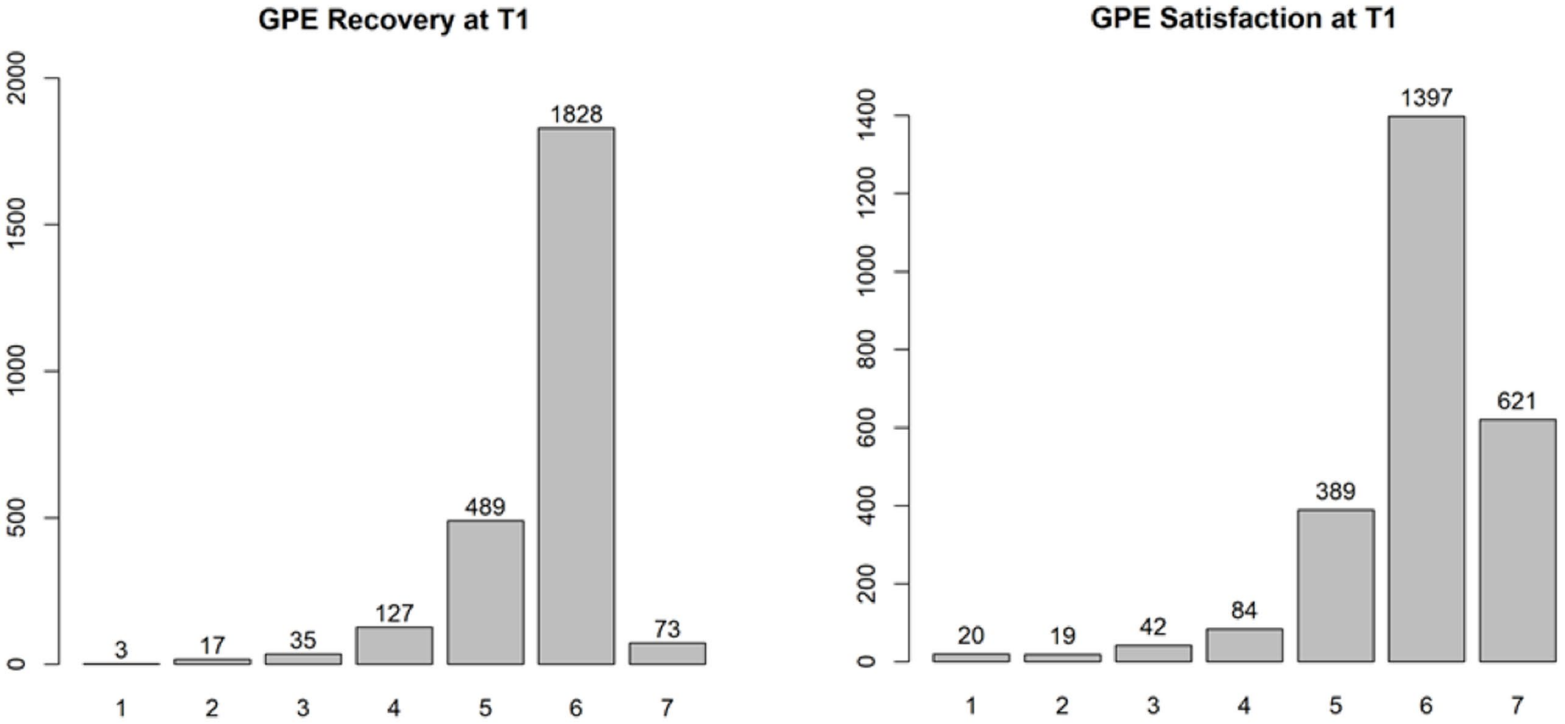
Results of GPE on the post-evaluation: recovery and satisfaction (T1).

Pain, sleep, activity, and MQS time were significant in the linear mixed model (*p* < 0.0001), and post hoc tests showed a significant decrease in pain and MQS from weeks 1 to 2, and from weeks 2 to 3. In addition, there was a significant increase in sleep quality and activity levels from weeks 1 to 2 and from weeks 2 to 3 (*p* < 0.0001). When looking at the results of the post-trial evaluation, we observed that all psychological measures decreased significantly after the trial period compared to the pre-trial findings (post hoc tests of the linear mixed model) (see Table 5). Interpreting the Dutch scores using the norms of the chronic pain population, several subscales clinically decreased after the completion of the trial period: SCL90 Depression, SCL90 Somatic Complaints, SCL90 Insufficiency, SCL90 Sensitivity, SCL90 Sleep problems, and the total score SCL90 Psychoneuroticism (see Table 6; bold for subscales with a significant clinical change during the trial period). Figure 3 presents the results of the GPE Recovery and Satisfaction questions. Of the patients, 71.1% (*n* = 1,828) answered “much improved” and 54.3% (*n* = 1,397) answered “very satisfied.”

**Table 5:** Comparison and evaluation of psychological measures pre- vs. post-evaluation (T0 vs. T1).

Psychological measures	Mean (SD) pre (T0)	Mean (SD) post (T1)	Estimate (95% CI)	*p*-value
SCL90 agoraphobia^a^	10.03 (15.33)	5.23 (10.30)	−4.78 [−5.54, −4.02]	**<0.001**
SCL90 anxiety^a^	18.33 (15.46)	8.69 (10.52)	−9.62 [−10.40, −8.84]	**<0.001**
SCL90 depression^a^	25.81 (18.67)	12.56 (13.37)	−13.24 [−14.21, −12.27]	**<0.001**
SCL90 somatic complaints^a^	37.41 (15.11)	17.90 (13.29)	−19.48 [−20.39, −18.58]	**<0.001**
SCL90 insufficiency^a^	35.61 (18.27)	19.09 (15.10)	−16.52 [−17.50, −15.53]	**<0.001**
SCL90 sensitivity^a^	13.18 (14.09)	6.64 (9.50)	−6.53 [−7.21, −5.86]	**<0.001**
SCL90 hostility^a^	11.53 (12.89)	5.32 (8.09)	−6.18 [−6.83, −5.52]	**<0.001**
SCL90 sleep problems^a^	61.13 (27.93)	31.51 (27.13)	−29.63 [−31.49, −27.76]	**<0.001**
SCL90 Psychoneuroticism^a^	22.58 (13.11)	11.24 (9.82)	−11.33 [−12.00, −10.65]	**<0.001**
SCL90 somatization^b^	23.47 (7.64)	9.45 (6.95)	−14.03 [−14.90, −13.16]	**<0.001**
SCL90 obsessive-compulsiveness^b^	15.94 (8.02)	6.95 (6.51)	−8.95 [−9.73, −8.18]	**<0.001**
SCL90 sensitivity^b^	8.11 (7.12)	3.24 (4.66)	−4.86 [−5.45, −4.26]	**<0.001**
SCL90 depression^b^	19.68 (11.27)	7.71 (8.46)	−11.95 [−13.02, −10.88]	**<0.001**
SCL90 anxiety^b^	10.17 (7.59)	3.74 (4.91)	−6.42 [−7.09, −5.75]	**<0.001**
SCL90 hostility^b^	5.13 (4.83)	1.81 (2.76)	−3.29 [−3.72, −2.87]	**<0.001**
SCL90 phobic anxiety^b^	5.67 (6.08)	2.72 (4.19)	−2.94 [−3.45, −2.44]	**<0.001**
SCL90 paranoid ideation^b^	3.69 (4.38)	1.78 (3.00)	−1.90 [−2.26, −1.53]	**<0.001**
SCL90 psychoticism^b^	5.10 (5.40)	2.08 (3.54)	−3.00 [−3.45, −2.55]	**<0.001**
SCL90 Global Severity Index^b^	1.20 (0.63)	0.49 (0.47)	−0.71 [−0.76, −0.65]	**<0.001**

Mean (SD) at T0 and T1. Estimated difference T1–T0 with 95% CI and *p*-value post hoc test of the linear mixed model.

a Dutch version and scoring of the SCL-90-R.

b French and German versions and scoring of the SCL-90-R. Values in bold indicate statistically significant differences.

**Table 6:** Comparison and evaluation of psychological measures pre- vs. post-evaluation (T0 vs. T1).

Psychological pre-evaluative screening	Interpretation pre (T0)	Interpretation post (T1)
SCL90 agoraphobia^a^	Average	Average
SCL90 anxiety^a^	Average	Average
**SCL90 depression** ^a^	**Above average**	**Below average**
**SCL90 somatic complaints** ^a^	**Above average**	**Below average**
**SCL90 insufficiency** ^a^	**Above average**	**Average**
**SCL90 sensitivity** ^a^	**Above average**	**Average**
SCL90 hostility^a^	Average	Average
**SCL90 sleep problems** ^a^	**Above average**	**Average**
**SCL90 Psychoneuroticism** ^a^	**Above average**	**Below average**

Interpretation norms chronic pain population.

a Dutch version and scoring of the SCL-90-R.

### 3.4 Multidisciplinary Pain Meeting (2) (T1)

After the trial and post-evaluation periods, a Multidisciplinary Pain Meeting (MAO) approved 92.5% of the records for permanent implants. The procedure was terminated for the remaining 7.5% of records. In 3% of cases (*n* = 97), the procedure was stopped during the trial period. Infection was a major point of interest during the trial period. Therefore, the cases that were stopped during the trial were further examined by screening the open textbox within the platform (where the physician could enter the reason for the premature ending of the procedure) for the word “infection.” For 26 of the 97 records (26.8%) “infection” was the reason for interrupting the trial period. Other reasons for interruption of the trial period were very broad, ranging from failure to obtain paresthesia in the proper dermatome, technical issues, fear of the patient for the observed paresthesia, insufficient clinical efficacy, and occurrence of bleeding during the electrode placement.

### 3.5 Yellow flags were predictive of less recovery and less satisfaction after trial (T1)

Logistic regression analyses of the Sub dataset showed no significant predictors of recovery. However, several significant predictors of satisfaction were observed: PCI Total Passive Coping, PCI Retreating, PCI Worrying, IAS Illness anxiety, and IAS Illness behavior (see Table 7). Ordinal regression analyses of the Sub dataset showed significant predictors for recovery: PCI Total Active Coping, IAS Illness anxiety, and IAS Illness behavior (see Table 8). Again, several significant predictors of satisfaction were observed: PCI Total Active Coping, PCI Pain transformation, PCI Distraction, IAS Illness anxiety, IAS Illness behavior, and SCL90 sleep problems^a^ (see Tables 2, 3, 5 for referencing).

**Table 7:** Predictive value of the pre-evaluative “yellow flags” on recovery and satisfaction after trial period (T1).

Predictors	Recovery			Satisfaction		
	OR	95% CI	Corrected *p*-value	OR	95% CI	Corrected *p*-value
PCI total active coping	1.010	[0.999, 1.020]	0.063	1.010	[0.999, 1.021]	0.066
PCI pain transforming	1.003	[0.996, 1.010]	0.599	1.007	[0.999, 1.014]	0.238
PCI distraction	1.008	[1.000, 1.016]	0.190	1.007	[0.999, 1.016]	0.238
PCI reducing demans	1.003	[0.997, 1.009]	0.599	1.000	[0.994, 1.006]	0.909
**PCI total passive coping**	**0.995**	**[0.986, 1.004]**	**0.302**	**0.985**	**[0.976, 0.995]**	**0.003**
**PCI retreating**	**1.000**	**[0.992, 1.007]**	**1.000**	**0.991**	**[0.984, 0.999]**	**0.046**
**PCI worrying**	**0.994**	**[0.986, 1.001]**	**0.316**	**0.988**	**[0.980, 0.996]**	**0.009**
PCI resting	0.999	[0.992, 1.006]	1.000	0.995	[0.988, 1.002]	0.168
**IAS illness anxiety**	**0.982**	**[0.965, 1.001]**	**0.121**	**0.967**	**[0.950, 0.985]**	**0.001**
**IAS illness behavior**	**1.003**	**[0.960, 1.047]**	**0.894**	**0.949**	**[0.905, 0.995]**	**0.028**
SCL90 agoraphobia^a^	0.999	[0.989, 1.011]	1.000	0.994	[0.984, 1.006]	1.000
SCL90 anxiety^a^	0.996	[0.985, 1.007]	1.000	0.992	[0.981, 1.003]	1.000
SCL90 depression^a^	0.995	[0.986, 1.004]	1.000	0.989	[0.980, 0.998]	0.172
SCL90 somatic complaints^a^	1.006	[0.995, 1.018]	1.000	1.000	[0.988, 1.013]	1.000
SCL90 insufficiency^a^	0.998	[0.989, 1.007]	1.000	0.998	[0.988, 1.008]	1.000
SCL90 sensitivity^a^	0.996	[0.985, 1.008]	1.000	0.993	[0.981, 1.006]	1.000
SCL90 hostility^a^	1.004	[0.991, 1.018]	1.000	0.995	[0.982, 1.009]	1.000
SCL90 sleep problems^a^	1.005	[0.999, 1.011]	1.000	1.005	[0.998, 1.011]	1.000
SCL90 Psychoneuroticism^a^	0.998	[0.985, 1.011]	0.708	0.991	[0.978, 1.005]	0.199
SCL90 somatization^b^	1.046	[1.001, 1.095]	0.352	1.020	[0.981, 1.063]	1.000
SCL90 obsessive-compulsiveness^b^	1.046	[1.002, 1.096]	0.352	1.013	[0.976, 1.054]	1.000
SCL90 sensitivity^b^	1.017	[0.971, 1.070]	1.000	0.992	[0.953, 1.035]	1.000
SCL90 depression^b^	1.011	[0.982, 1.042]	1.000	0.991	[0.966, 1.018]	1.000
SCL90 anxiety^b^	1.016	[0.973, 1.066]	1.000	0.985	[0.950, 1.025]	1.000
SCL90 hostility^b^	1.029	[0.960, 1.115]	1.000	0.996	[0.940, 1.063]	1.000
SCL90 phobic anxiety^b^	1.010	[0.958, 1.072]	1.000	0.982	[0.939, 1.031]	1.000
SCL90 paranoid ideation^b^	0.992	[0.926, 1.073]	1.000	0.960	[0.906, 1.025]	1.000
SCL90 psychoticism^b^	0.983	[0.933, 1.045]	1.000	0.951	[0.910, 0.999]	0.399
SCL90 Global Severity Index^b^	1.321	[0.774, 2.382]	0.319	0.902	[0.575, 1.462]	0.665
MQS day 0	1.010	[0.995, 1.027]	0.202	1.009	[0.993, 1.026]	0.296

OR with 95% CI from the logistic regression model and *p*-value corrected for multiple testing. Statistically significant differences are indicated in bold.

a Dutch version and scoring of the SCL-90-R.

b French and German version and scoring of the SCL-90-R. Values in bold indicate statistically significant differences.

**Table 8:** Predictive value of the pre-evaluative “yellow flags” on recovery and satisfaction after trial period (T1).

Predictors	Recovery			Satisfaction		
	OR	95% CI	Corrected *p*-value	OR	95% CI	Corrected *p*-value
PCI total active coping	1.006	[1.001, 1.012]	**0.033**	1.010	[1.005, 1.016]	**<0.001**
PCI pain transforming	1.005	[1.001, 1.009]	0.066	1.007	[1.004, 1.011]	**<0.001**
PCI distraction	1.004	[1.000, 1.009]	0.153	1.009	[1.005, 1.013]	**<0.001**
PCI reducing demans	1.000	[0.997, 1.003]	0.911	0.999	[0.996, 1.002]	0.406
PCI total passive coping	0.997	[0.992, 1.002]	0.208	0.996	[0.992, 1.001]	0.088
PCI retreating	0.999	[0.995, 1.003]	0.795	0.999	[0.995, 1.003]	0.857
PCI worrying	0.997	[0.993, 1.001]	0.507	0.996	[0.992, 0.999]	0.071
PCI resting	0.998	[0.995, 1.002]	0.795	0.999	[0.995, 1.002]	0.857
**IAS illness anxiety**	**0.980**	**[0.970**,**0.991]**	**<0.001**	0.975	[0.966, 0.985]	**<0.001**
**IAS illness behavior**	**0.967**	**[0.943**,**0.991]**	**0.007**	0.965	[0.945, 0.986]	**0.001**
SCL90 agoraphobia^a^	0.994	[0.988, 1.001]	0.573	0.998	[0.992, 1.003]	1.000
SCL90 anxiety^a^	0.996	[0.989, 1.002]	0.901	0.995	[0.989, 1.001]	0.528
SCL90 depression^a^	0.995	[0.990, 1.001]	0.575	0.995	[0.990, 0.999]	0.166
SCL90 somatic complaints^a^	0.994	[0.988, 1.001]	0.575	1.000	[0.994, 1.005]	1.000
SCL90 insufficiency^a^	0.998	[0.992, 1.003]	1.000	1.000	[0.996, 1.005]	1.000
SCL90 sensitivity^a^	1.000	[0.993, 1.008]	1.000	0.997	[0.991, 1.004]	1.000
SCL90 hostility^a^	1.000	[0.992, 1.008]	1.000	0.998	[0.991, 1.004]	1.000
SCL90 sleep problems^a^	1.002	[0.999, 1.006]	0.901	1.005	[1.002, 1.008]	**0.010**
SCL90 Psychoneuroticism^a^	0.996	[0.989, 1.004]	0.297	0.997	[0.990, 1.003]	0.348
SCL90 somatization^b^	1.010	[0.990, 1.031]	1.000	1.017	[0.999, 1.036]	0.582
SCL90 obsessive-compulsiveness^b^	1.009	[0.990, 1.029]	1.000	1.011	[0.993, 1.028]	1.000
SCL90 sensitivity^b^	1.006	[0.984, 1.028]	1.000	1.006	[0.986, 1.026]	1.000
SCL90 depression^b^	1.002	[0.988, 1.016]	1.000	1.000	[0.988, 1.013]	1.000
SCL90 anxiety^b^	1.000	[0.980, 1.021]	1.000	1.001	[0.983, 1.019]	1.000
SCL90 hostility^b^	1.029	[0.995, 1.064]	0.868	1.016	[0.987, 1.046]	1.000
SCL90 phobic anxiety^b^	0.993	[0.968, 1.018]	1.000	1.001	[0.978, 1.024]	1.000
SCL90 paranoid ideation^b^	1.007	[0.971, 1.043]	1.000	1.000	[0.969, 1.033]	1.000
SCL90 psychoticism^b^	0.989	[0.961, 1.017]	1.000	0.994	[0.968, 1.020]	1.000
SCL90 Global Severity Index^b^	1.056	[0.822, 1.357]	0.668	1.079	[0.864, 1.347]	0.501
MQS day 0	1.004	[0.995, 1.012]	0.419	1.003	[0.995, 1.010]	0.472

OR with 95% CI from the ordinal regression model and *p*-value corrected for multiple testing. Statistically significant differences are indicated in bold.

a Dutch version and scoring of the SCL-90-R.

b French and German versions and scoring of the SCL-90-R. Values in bold indicate statistically significant differences.

### 3.6 Outcome measures during chronic follow-up (6 months follow-up after permanent implant)

**Figure 4:**
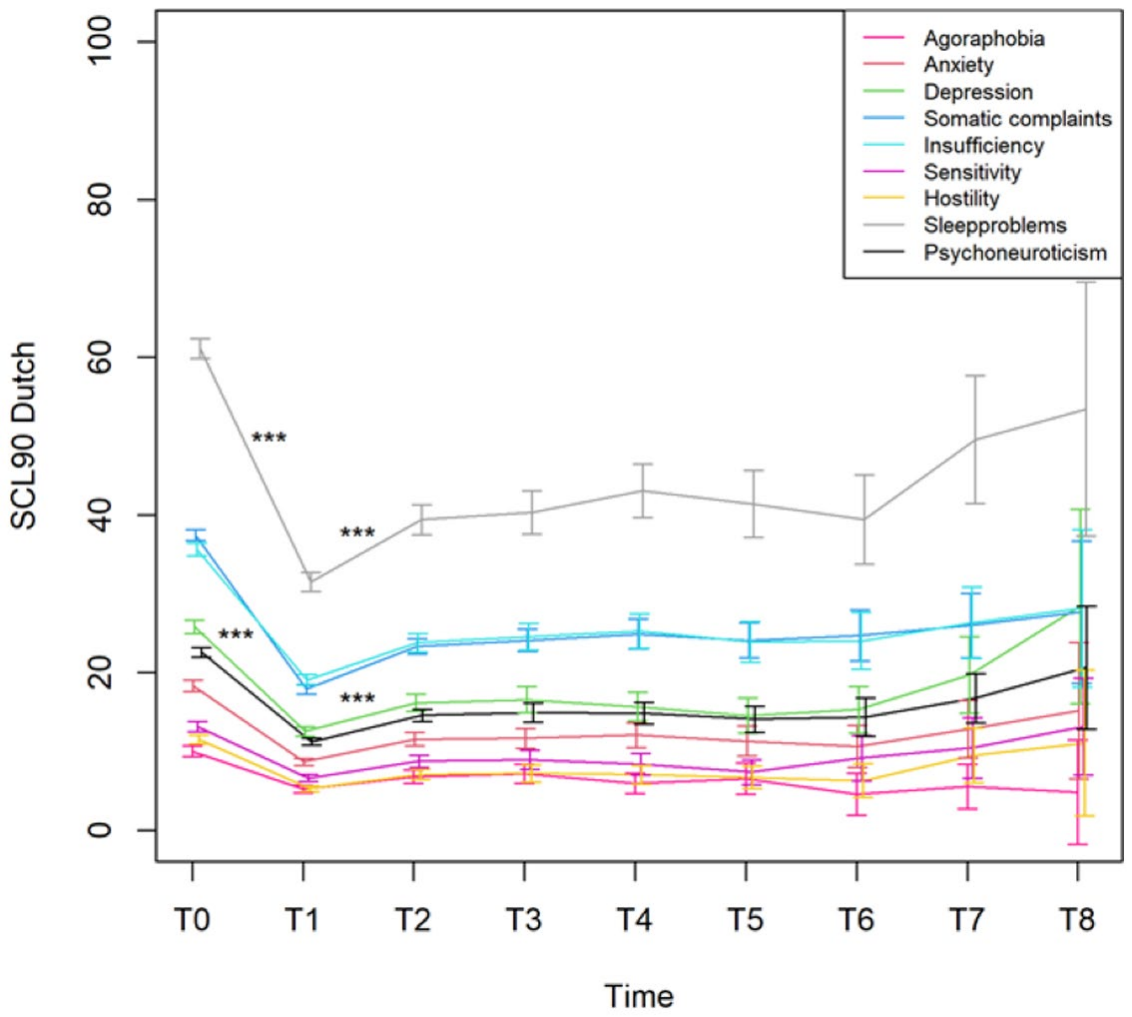
Mean with 95% CI of the total score Psychoneuroticism and the subscales of the Dutch version of the SCL90 from the pre (T0)- and post-evaluation (T1) and the seven follow-up time points (T2–T8). ^***^p-value <0.001.

To see if outcome measures evolve, the main effect of time was first considered in the linear mixed model. The *p*-value for this main effect was significant (*p* < 0.001) for all variables (even after Bonferroni–Holm correction for the subscales), except for PCI Pain transformation (*p* = 0.187) (Figure 4). Also, for PCI Total Active Coping time was significant (*p* = 0.04).

The changes from time point T1 (post-evaluation) to consecutive time point T2 (after 6 months) on the SCL90 were all significant (all with a *p*-value <0.001, except for the French and German versions of the SCL90 Paranoid ideation that had a *p* = 0.006). All other changes at the different time points were not statistically significant.

**Figure 5:**
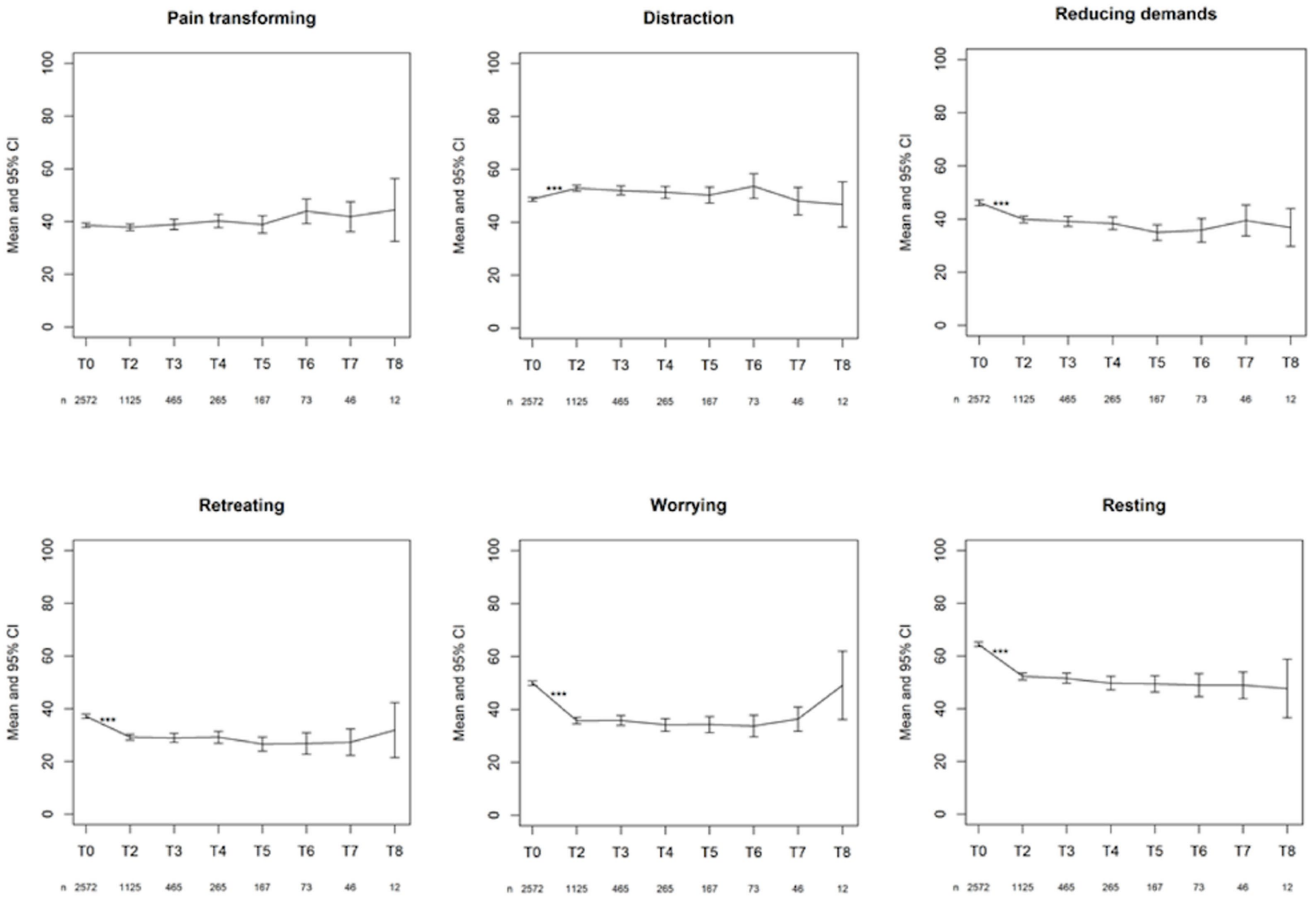
First line: mean with 95% CI of the three active coping subscales. Second line: mean with 95% CI of the three passive coping subscales. ^***^p-value <0.001.

**Figure 6:**
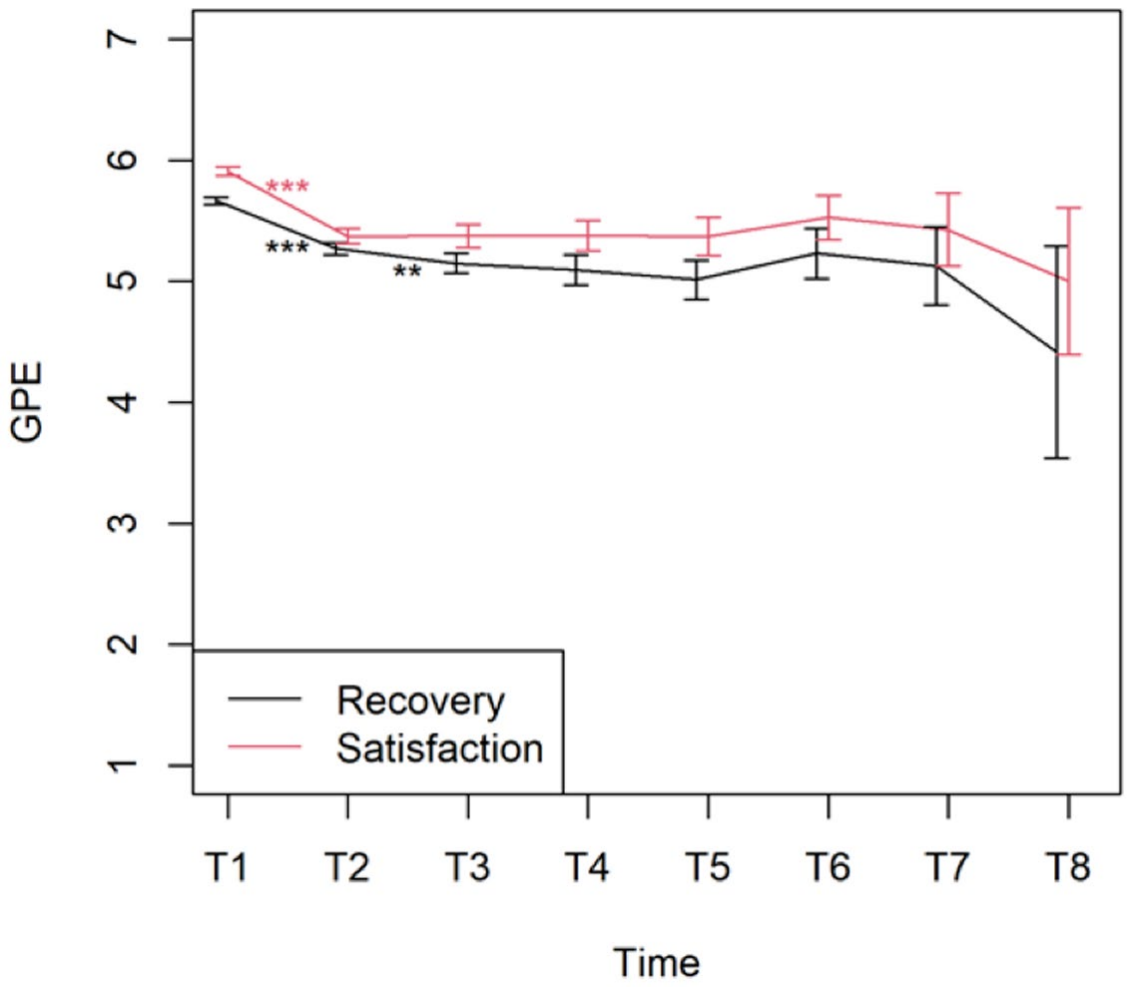
Mean with 95% CI of GPE recovery and satisfaction. ^***^p-value <0.001 and ^**^p-value <0.01.

The changes from time point T1 (post-evaluation) to the consecutive time point T2 (after 6 months) on the PCI subscales were all significant (*p* < 0.001), except for Total Active Coping (*p* = 1.000). None of the changes between the other time points were significant (Figure 5). Figure 6 displays the changes from time point T1 (post-evaluation) to consecutive time point T2 (after 6 months) on the GPE Recovery and Satisfaction subscales. These changes were statistically significant (p < 0.001). The change in recovery from T2 (after 6 months) to the consecutive time point T3 (after 1 year) was also significant (*p* = 0.003). All other changes between subsequent time points were not significant.

**Figure 7:**
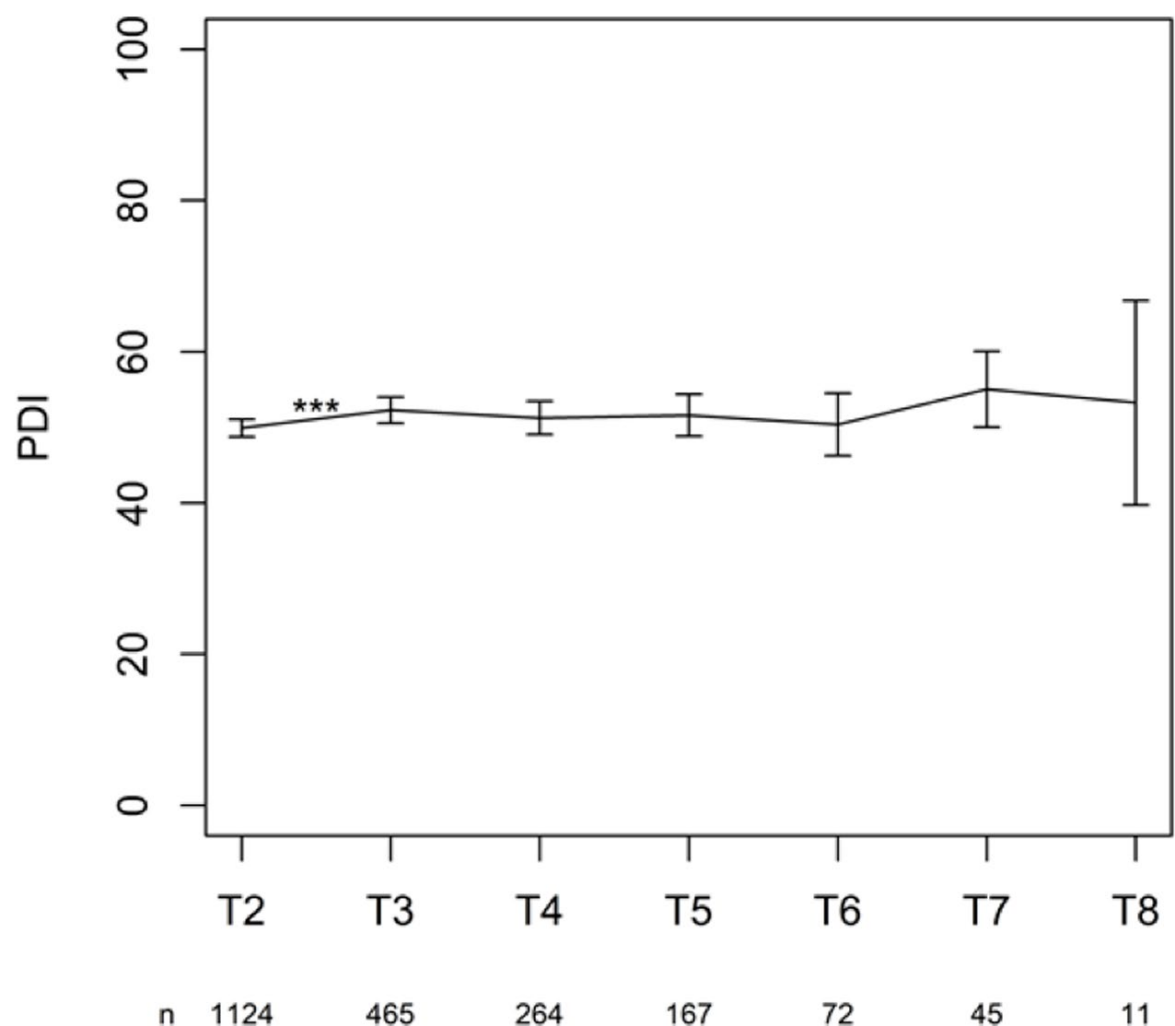
Mean with 95% CI of PDI (in %). ^***^p-value <0.001.

The change in PDI from T2 (after 6 months) to the consecutive time point T3 (after 1 year) on the PDI was significant (*p* < 0.001). None of the other changes between time points reached significance (Figure 7).

### 3.7 Are yellow flags predictive of lower functioning after 6 months (T2) and 1.5 years (T4)?

Logistic regression analyses of the entire dataset showed significant predictors for disability after 6 months: PCI Total Passive Coping, PCI Retreating, PCI Worrying, PCI Resting, IAS Illness behavior, SCL90 Agoraphobia^a^, SCL90 Depression^a^, SCL90 Somatic Complaints^a^, SCL90 Insufficiency^a^, SCL90 Sensitivity^a^, SCL90 Sleep problems^a^, SCL90 Psychoneuroticism^a^, SCL90 Somatization^b^, SCL90 Anxiety^b^, SCL90 Global Severity Index^b^ and MQS day 0 (Table 9). After 1.5 years, the analyses showed two significant predictors for disability: PCI Resting and SCL90 Global Severity Index^†^. Linear regression analyses of the full dataset showed significant predictors for disability after 6 months (Table 10): PCI Total Passive Coping, PCI Retreating, PCI Worrying, PCI Resting, IAS Illness anxiety, IAS Illness behavior, SCL90 Psychoneuroticism^a^ and all the Dutch subscales of the SCL90, SCL90 Somatization^b^, SCL90 Depression^b^, SCL90 Anxiety^b^, SCL90 Phobic anxiety^b^, SCL90 Global Severity Index^b^ and MQS day 0. The analyses showed the following significant predictors of disability after 1.5 years: PCI Total Passive Coping, PCI Resting, IAS Illness behavior, SCL90 Agoraphobia^a^, SCL90 Somatic Complaints^a^, SCL90 Insufficiency^a^, SCL90 Psychoneuroticism^a^, SCL90 Obsessive-compulsiveness^b^, SCL90 Global Severity Index^b^ and MQS day 0.

**Table 9:** Predictive value of the pre-evaluative “yellow flags” on disability after 6 months (T2) and 1.5 years (T4).

Predictors	Follow-up 1: after 6 months (T2)			Follow-up 3: after 1.5 years (T4)		
	OR	95% CI	Corrected *p*-value	OR	95% CI	Corrected *p*-value
PCI total active coping	0.998	[0.990, 1.006]	0.575	1.004	[0.986, 1.022]	0.693
PCI pain transforming	0.999	[0.994, 1.005]	1.000	1.011	[0.999, 1.023]	0.187
PCI distraction	0.998	[0.992, 1.005]	1.000	0.999	[0.987, 1.012]	0.911
PCI reducing demans	0.999	[0.995, 1.004]	1.000	0.995	[0.985, 1.005]	0.612
PCI total passive coping	1.023	[1.015, 1.031]	**<0.001**	1.016	[1.000, 1.033]	0.057
PCI retreating	1.014	[1.008, 1.020]	**<0.001**	1.014	[1.001, 1.028]	0.072
PCI worrying	1.012	[1.006, 1.018]	**<0.001**	1.001	[0.988, 1.013]	0.930
PCI resting	1.016	[1.010, 1.022]	**<0.001**	1.019	[1.007, 1.032]	**0.006**
IAS illness anxiety	1.013	[0.998, 1.029]	0.096	0.979	[0.946, 1.013]	0.445
IAS illness behavior	1.122	[1.082, 1.165]	**<0.001**	1.035	[0.959, 1.119]	0.445
SCL90 agoraphobia^a^	1.016	[1.007, 1.026]	**0.003**	1.016	[0.998, 1.035]	0.625
SCL90 anxiety^a^	1.006	[0.997, 1.015]	0.222	1.000	[0.983, 1.018]	1.000
SCL90 depression^a^	1.016	[1.008, 1.023]	**<0.001**	1.007	[0.992, 1.022]	1.000
SCL90 somatic complaints^a^	1.020	[1.011, 1.030]	**<0.001**	1.011	[0.994, 1.029]	1.000
SCL90 insufficiency^a^	1.020	[1.012, 1.028]	**<0.001**	1.018	[1.003, 1.034]	0.125
SCL90 sensitivity^a^	1.014	[1.004, 1.024]	**0.019**	1.013	[0.992, 1.036]	1.000
SCL90 hostility^a^	1.009	[0.998, 1.020]	0.222	1.014	[0.989, 1.040]	1.000
SCL90 sleep problems^a^	1.008	[1.003, 1.013]	**0.004**	0.998	[0.989, 1.007]	1.000
SCL90 Psychoneuroticism^a^	1.022	[1.011, 1.033]	**<0.001**	1.016	[0.994, 1.039]	0.155
SCL90 somatization^b^	1.048	[1.013, 1.086]	**0.049**	0.970	[0.837, 1.112]	0.659
SCL90 obsessive-compulsiveness^b^	1.030	[0.997, 1.064]	0.377	0.892	[0.740, 1.028]	0.582
SCL90 sensitivity^b^	1.022	[0.985, 1.061]	0.653	0.738	[0.488, 0.950]	0.129
SCL90 depression^b^	1.028	[1.004, 1.053]	0.152	0.921	[0.807, 1.029]	0.582
SCL90 anxiety^b^	1.064	[1.024, 1.109]	**0.011**	0.852	[0.671, 1.005]	0.405
SCL90 hostility^b^	1.025	[0.965, 1.090]	0.653	0.764	[0.501, 1.065]	0.582
SCL90 phobic anxiety^b^	1.051	[1.005, 1.101]	0.182	0.756	[0.507, 0.990]	0.328
SCL90 paranoid ideation^b^	1.042	[0.976, 1.113]	0.653	0.770	[0.497, 1.103]	0.582
SCL90 psychoticism^b^	1.044	[0.989, 1.104]	0.475	0.794	[0.570, 1.030]	0.500
SCL90 Global Severity Index^b^	1.719	[1.112, 2.711]	**0.014**	0.054	[0.001, 0.793]	**0.031**
MQS day 0	1.013	[1.001, 1.026]	**0.037**	1.026	[0.998, 1.055]	0.067

OR with 95% CI from the logistic regression model and *p*-value corrected for multiple testing. Statistically significant differences are indicated in bold.

a Dutch version and scoring of the SCL-90-R.

b French and German version and scoring of the SCL-90-R. Values in bold indicate statistically significant differences.

**Table 10:** Predictive value of the pre-evaluative “yellow flags” on disability after 6 months (T2) and 1.5 years (T4).

Predictors	Follow-up 1: after 6 months (T2)			Follow-up 3: after 1.5 years (T4)		
	Est.	95% CI	Corrected *p*-value	Est.	95% CI	Corrected *p*-value
PCI total active coping	−0.036	[−0.118, 0.046]	0.388	−0.018	[−0.179, 0.143]	0.826
PCI pain transforming	0.014	[−0.041, 0.069]	1.000	0.066	[−0.038, 0.170]	0.634
PCI distraction	−0.061	[−0.126, 0.003]	0.189	−0.039	[−0.155, 0.077]	0.647
PCI reducing demans	−0.006	[−0.051, 0.040]	1.000	−0.046	[−0.138, 0.046]	0.647
PCI total passive coping	0.317	[0.246, 0.388]	**<0.001**	0.200	[0.056, 0.344]	**0.007**
PCI retreating	0.182	[0.125, 0.240]	**<0.001**	0.101	[−0.016, 0.219]	0.183
PCI worrying	0.182	[0.124, 0.240]	**<0.001**	0.070	[−0.043, 0.184]	0.225
PCI resting	0.227	[0.174, 0.279]	**<0.001**	0.221	[0.118, 0.325]	**<0.001**
IAS illness anxiety	0.228	[0.074, 0.383]	**0.004**	−0.143	[−0.452, 0.166]	0.363
IAS illness behavior	1.540	[1.202, 1.878]	**<0.001**	0.824	[0.137, 1.510]	**0.038**
SCL90 agoraphobia^a^	0.225	[0.138, 0.312]	**<0.001**	0.251	[0.098, 0.405]	**0.011**
SCL90 anxiety^a^	0.148	[0.060, 0.235]	**0.002**	0.128	[−0.031, 0.288]	0.243
SCL90 depression^a^	0.213	[0.143, 0.284]	**<0.001**	0.146	[0.013, 0.280]	0.157
SCL90 somatic complaints^a^	0.307	[0.220, 0.395]	**<0.001**	0.237	[0.082, 0.392]	**0.017**
SCL90 insufficiency^a^	0.293	[0.223, 0.362]	**<0.001**	0.201	[0.074, 0.328]	**0.015**
SCL90 sensitivity^a^	0.192	[0.095, 0.288]	**<0.001**	0.186	[−0.008, 0.380]	0.239
SCL90 hostility^a^	0.144	[0.041, 0.247]	**0.006**	0.200	[−0.025, 0.424]	0.243
SCL90 sleep problems^a^	0.099	[0.052, 0.147]	**<0.001**	−0.008	[−0.091, 0.074]	0.844
SCL90 Psychoneuroticism^a^	0.323	[0.223, 0.423]	**<0.001**	0.264	[0.070, 0.458]	**0.008**
SCL90 somatization^b^	0.578	[0.230, 0.926]	**0.010**	−0.793	[−1.711, 0.125]	0.341
SCL90 obsessive-compulsiveness^b^	0.384	[0.046, 0.723]	0.131	−1.246	[−1.976, −0.515]	**0.023**
SCL90 sensitivity^b^	0.318	[−0.071, 0.706]	0.325	−1.602	[−2.752, −0.451]	0.078
SCL90 depression^b^	0.330	[0.088, 0.572]	**0.046**	−0.718	[−1.455, 0.019]	0.277
SCL90 anxiety^b^	0.647	[0.263, 1.030]	**0.009**	−1.172	[−2.088, −0.256]	0.111
SCL90 hostility^b^	0.192	[−0.447, 0.832]	0.554	−1.162	[−3.463, 1.139]	0.591
SCL90 phobic anxiety^b^	0.649	[0.189, 1.110]	**0.041**	−1.606	[−3.122, −0.089]	0.237
SCL90 paranoid ideation^b^	0.521	[−0.165, 1.207]	0.325	−1.623	[−4.191, 0.946]	0.591
SCL90 psychoticism^b^	0.609	[0.047, 1.172]	0.136	−1.056	[−2.913, 0.800]	0.591
SCL90 Global Severity Index^b^	6.608	[2.136, 11.081]	**0.004**	−18.910	[−31.340, −6.480]	**0.006**
MQS day 0	0.173	[0.052, 0.295]	**0.005**	0.320	[0.082, 0.559]	**0.009**

OR with 95% CI from the linear regression model and *p*-value corrected for multiple testing. Statistically significant differences are indicated in bold.

a Dutch version and scoring of the SCL-90-R.

b French and German version and scoring of the SCL-90-R. Values in bold indicate statistically significant differences.

## 4 Discussion

### 4.1 Principal results

#### 4.1.1 Participants

This large real-world dataset included 7,304 records from 6,170 patients, with a mean age of 57.7 years and a predominance of females. This finding was in accordance with those of epidemiological studies indicating a higher prevalence of chronic pain in women. A small majority of the records were concerned with the replacement procedures. In both the first implant and replacement implant procedures, most records indicated the presence of PSPS type 2 (failed back surgery and failed neck surgery syndromes) as the reason for chronic pain. The interpretation of data was complicated because of the use of questionnaires in different languages. Almost three-fourths of the patients opted to complete the questionnaires in Dutch. With repeated activation of the module for chronic follow-up, the number of patients who completed the questionnaire decreased. Upon evaluation of the current procedure, it seems logical that the completion of the questionnaires with the chronic follow-up module should be either mandatory or linked to a motivating experience, to optimize the long-term monitoring of treatment outcomes.

#### 4.1.2 Multidisciplinary Pain Meeting (T0)

After multidisciplinary screening, the Multidisciplinary Pain Meeting approved 99% of the trial implant records. The procedure was terminated in only 1% of the records. Statistical analysis showed a significant difference in Psychoneuroticism (measured by the SCL-90-R, Dutch version) between the group that stopped before the initiation of the trial and the group that continued into the trial period. Patients not approved by the multidisciplinary team to start the trial period showed an increased level of general physical and psychological dysfunction compared to patients who moved on to the trial. Interpreting the Dutch scores using the norms of the population with chronic pain, their general level of physical and psychological dysfunction was also statistically and clinically higher. Although the group of patients not approved by the multidisciplinary team was small in absolute number (*n* = 31) compared to the group that continued the trial period (*n* = 2,569), the observed differences clearly indicate the added value of the extended multidisciplinary screening of patients that was implemented.

#### 4.1.3 Outcome measures after the 3 weeks trial period

During the 3 weeks trial period, there was a statistically significant improvement in pain, activity, and sleep quality. The intake of pain medications also significantly decreased during the trial. Importantly, we only obtained data from the trial period. These data allowed us to make statements about the evolution of pain, activity levels, and sleep quality during the trial period. As we currently miss data points before the initiation of the trial period, we cannot draw conclusions about the evolution of these variables before the start of stimulation. This concern is further described in the section “Limitations.” For the SCL-90-R, we compared the results before and after the trial period. A statistical analysis showed significant improvements in all variables measured using the SCL-90-R at the completion of the trial period. A significant difference was observed in the Dutch, French, and German versions. The patients reported fewer symptoms of agoraphobia, anxiety, depression, and sleep disorders. They experienced fewer cognitive problems and fewer deficits in thinking and behavior. This could be partially explained by the decreased intake of pain medication, which has side effects on cognitive function. They felt less uneasy with themselves in relation to other people and reported fewer feelings of hostility. Their levels of somatic complaints declined compared to those before the trial. The general levels of physical and psychological dysfunction improved. When the scores of the Dutch version were interpreted using the norms of the chronic pain population, patients showed clinical improvements in every variable except Agoraphobia, Anxiety, and Hostility. However, even these three subscales halved compared to the trial period’s start. Most patients reported that their complaints improved significantly, and they felt very satisfied with the clinical results of SCS during the trial period.

#### 4.1.4 Second Multidisciplinary Pain Meeting (T1)

After the 3 weeks trial period and completion of the post-trial evaluation, the Multidisciplinary Pain Meeting approved 92.5% of the records for a permanent implant. Some patients (*n* = 97) ended the trial prematurely. In 26 of these cases, infection was identified as the reason for such premature termination of the trial. This could be interpreted as if the infection rate in this large real-life database amounts to 2% of the total number of included cases, which would be a very low rate of infection (North et al., 2020; Ege et al., 2023). However, other factors need to be considered. Infection can also occur at a later stage, such as immediately after permanent implantation of the battery or infections can occur at a much later stage. The 2% only relates to the occurrence of infection during the 3 weeks trial period.

#### 4.1.5 Yellow flags are predictive of less recovery and less satisfaction after trial (T1)

The statistical analysis showed differences in the predictive value of yellow flags for the degrees of recovery and satisfaction after the trial period. Logistic regression analysis showed the predictive value of pain coping strategies and illness attitudes for the degree of satisfaction after the trial period. Ordinal regression analysis showed the predictive value of pain-coping strategies and illness attitudes for both the degrees of recovery and satisfaction after the trial period. Feeling recovered and satisfied appear to be two different matters.

Pain coping strategies predict how recovered and satisfied patients feel with the results of the trial period. Patients who used more passive pain-coping strategies seemed less satisfied after the trial period (logistic regression analysis). Patients using more active pain-coping strategies seemed to experience greater recovery and satisfaction after the trial period (ordinal regression analysis). The way a patient thinks, feels, and behaves in relation to pain influences the improvement and satisfaction after a trial period of neuromodulation.

Illness attitudes are predictive of how recovered and satisfied a patient felt with the results of the trial period. Patients experiencing more symptoms of illness anxiety (fears, attitudes, and beliefs associated with hypochondrial concerns) and illness behavior seemed to experience less recovery (ordinal regression analysis) and less satisfaction after the trial period (logistic and ordinal regression analyses). The way a patient thinks, feels, and behaves in relation to their health and illness influences their ability to experience improvement and satisfaction after a trial period of neuromodulation.

#### 4.1.6 Outcome measures chronic follow-up

The statistical analysis revealed a significant decrease during the trial period for all variables measured by the SCL-90-R (Dutch, French, and German versions). After permanent implantation, there was a statistically significant increase in all the variables (Dutch, French, and German versions) compared to what was reported after the end of the trial period. Six months later, the patients again reported higher physical and psychological dysfunctions compared to immediately after the termination of the trial period. However, at the following time points, there was no longer a statistically significant difference between the follow-up questionnaires; beyond the 6 months’ time point, physical and psychological dysfunction no longer evolved to be statistically significant. These results indicate that the initially observed improvement in physical and psychological dysfunction does not seem to hold in the long term. Furthermore, physical and psychological dysfunction increased again during the first 6 months and remained relatively stable afterwards.

There was a statistically significant decrease in all passive pain coping strategies during the first 6 months after permanent implantation. Patients have a lower need to retreat themselves, worry, and catastrophize, and do not need as much rest as before. However, active pain coping strategies have shown mixed results. There was a statistically significant increase in the use of distraction-oriented strategies during the first 6 months after permanent implantation. There was a statistically significant decrease of reducing demands despite pain during the first 6 months after the permanent implant. Pain-transforming strategies remained stable during the first 6 months after permanent implantation. There was no statistically significant difference between the other chronic follow-up questionnaires, indicating that pain-coping behavior did not evolve significantly. Thus, the results indicate that treatment with SCS changes patients’ pain-coping strategies in a constructive manner. Probably, these changes could be further enhanced if we would actively coach the patients on this subject.

Patients continued to feel recovered and satisfied after their permanent implant; however, there was a statistically significant decrease in their degree of recovery and satisfaction from the post-evaluation period to the first follow-up 6 months later. The decrease in the degree of recovery from the first follow-up after 6 months to the second follow-up after 1 year was also statistically significant. There was no statistically significant difference between the other chronic follow-up questionnaires, indicating that the degree of recovery and satisfaction did not evolve in a statistically significant manner. Thus, patients seem to lose their initial degree of recovery and satisfaction shortly after a permanent implant but still feel relatively recovered and satisfied with their SCS treatment.

The statistical analysis showed a significant increase in disability due to pain from the first follow-up at 6 months to the second follow-up 1 year after permanent implantation. There was no statistically significant difference between the other chronic follow-up questionnaires, indicating that the level of disability due to pain no longer evolved in a statistically significant way.

In conclusion, the results of the chronic follow-up questionnaires in our study were mixed. Some outcome variables were in line with the initial results of the 3 weeks trial period; other outcome variables did not further improve in the long term, and some variables even deteriorated again. During the first 6 months after permanent implantation, pain-related physical and psychological dysfunction and disability increased; on the other hand, pain-coping strategies evolved constructively. Patients felt relatively recovered and satisfied with their treatment; however, we observed over time a certain decline in this satisfaction. It should be mentioned that most of the included patients are suffering from failed back and failed neck surgery syndromes (nowadays categorized as PSPS type 2 syndromes) since this is the only indication reimbursed in Belgium under the current legislation. Other neuropathic pain conditions are perhaps more suitable for long-term treatment with invasive neuromodulation, but this can currently not be performed in Belgium due to legislative restrictions, so such indications are not present in our database.

#### 4.1.7 Yellow flags are predictive of lower functioning after 6 months (T2) and 1.5 years (T4)

Pain coping strategies predict how disabled a patient feels 6 months and 1.5 years after permanent implantation. Patients using more passive pain coping strategies seemed to feel more disabled after 6 months (logistic and linear regression analysis) and 1.5 years (linear regression analysis).

Illness attitudes are predictive of how disabled a patient feels 6 months and 1.5 years after a permanent implant. Patients experiencing more symptoms of illness anxiety (fear, attitudes, and beliefs associated with hypochondriacal concerns) seemed more disabled after 6 months (linear regression analysis). Patients showing more illness behavior seemed to feel more disabled after 6 months (logistic and linear regression analysis) and 1.5 years (linear regression analysis).

General physical and psychological dysfunction predicts how disabled a patient feels 6 months and 1.5 years after permanent implantation. Patients experiencing more physical and psychological dysfunction seemed to feel more disabled after 6 months (logistic and linear regression analysis) and 1.5 years (linear regression analysis), which was observed in the Dutch version of the SCL-90-R. In contrast, in the French and German versions of the SCL-90-R, patients experiencing more physical and psychological dysfunction seemed less disabled after 1.5 years (linear regression analysis).

Pain medication intake at the onset of the trial period appeared to be predictive of how disabled a patient felt 6 months and 1.5 years after permanent implantation. Patients with higher baseline doses of pain medication seemed to feel more disabled after 6 months (logistic and linear regression analyses) and after 1.5 years (linear regression analysis).

In conclusion, pain coping strategies, illness attitudes, general physical and psychological dysfunctioning, and even the intake of pain medication at the onset of the trial period (or “yellow flags”) predict how disabled a patient will feel after 6 months and 1.5 years after obtaining a permanent implant.

### 4.2 Comparison with previous findings

Several authors have highlighted the importance of preimplantation psychosocial variables in the treatment of SCS (Celestin et al., 2009). Psychological screening for “yellow flags” is a common practice in treatment with SCS. The results of this data analysis are consistent with a systematic review by Celestin et al. (2009) on the relationship between presurgical and preimplantation variables and treatment outcomes. They found a positive relationship between one or more psychological factors and poor treatment outcomes. Pretreatment somatization, depression, anxiety, and poor coping were the most predictive factors for reduced benefits from surgery or SCS. Our results also indicated that pain-coping strategies and illness attitudes (including somatization) predicted how recovered and satisfied a patient felt after the trial period. Pain coping strategies, illness attitudes, and general physical and psychological dysfunction predicted how disabled a patient feels 6 months and 1.5 years after permanent implantation. The subscales investigating anxiety and depression predict how disabled a patient will feel in the long term. This confirms the importance of pre-implantation psychological screening. In addition, every candidate for the first implant was psychologically screened. Patients in whom poor results were predicted did not continue the trial period. Our results also showed a difference in general pre-implantation physical and psychological dysfunction between eligible and non-eligible patients.

Our results show that the initial improvement in physical and psychological dysfunction during the trial period did not continue after permanent implantation. Six months after permanent implantation, patients reported higher physical and psychological dysfunction compared to immediately after the trial period. These results demonstrated the importance of sustained multidisciplinary follow-up, including psychological follow-up, after permanent implantation. Molloy et al. (2006) examined the value of intensive cognitive-behavioral pain management in addition to SCS. Their results support the idea that combined somatic and psychosocial interventions achieve better outcomes than single therapies. We emphasize that psychological evaluation is essential before initiating SCS treatment. Therefore, psychosocial factors can be identified and modified to improve the long-term effects of SCS.

As the conditions and design of the Neuro-Pain^®^ platform are defined in a Royal Decree (R Core Team, 2021), modifications to the system and questionnaires are only possible after reapplication by the Belgian Federal Government. As chronic follow-up questionnaires were added a few months after the start of the new reimbursement legislation, the PDI was not part of the pre- and post-evaluation psychological inventory. Currently, we only have PDI data from the chronic follow-up period. Therefore, we could not make statements about the effects of treatment on the patients’ disabilities. The literature describes guidelines for the interpretation of change in scores on the PDI (Soer et al., 2012). In patients with chronic low back pain, changes can be considered clinically important when the PDI score decreases from 8.5 to 9.5 points. Beemster et al. (2018) added that the interpretation of the change score is baseline specific. They retrospectively investigated the data of patients with chronic musculoskeletal pain after vocational rehabilitation. The higher the initial disability, the greater the room for improvement. They advise the following cut-off scores to decide if a PDI change score is clinically relevant: patients with a PDI baseline score of ≤27 should decrease minimal 7 points, patients with a baseline score between 28 and 42 should decrease minimal 15 points, and patients with a baseline score ≥43 should decrease minimal 20 points. In future system modifications, it is recommended that the PDI be added to the pre- and post-evaluation psychological inventory. Thus, conclusions can be drawn regarding the clinical significance of the evolution of PDI scores.

Hush et al. (2009) conducted a qualitative study on recovery in patients with chronic back pain using semi-structured interviews with focus groups. The authors investigated the meaning of “recovery” since patients sometimes report feeling much better, despite relatively unchanged outcome measures. Participants completed a pain scale and a disability questionnaire. Their self-rated recovery status did not seem to correspond to their level of pain or disability. Pain was not a reliable indicator of recovery. Cognitive appraisal of the impact of their symptoms on their ability to complete meaningful daily activities and fulfill social roles appears to play an important role in feeling recovered. Cognitive-behavioral strategies to manage pain, and even minimal pain, have been reported as major contributing factors to feeling more competent in dealing with pain. Changes in identity due to pain, grief, and loss are essential processes that patients must deal with. The authors propose patient-specific outcome measures as an interesting alternative to fixed items in capturing the highly individual concept of “recovery.” Doleys et al. (2006) reported that 25%–50% of patients with a successful trial period lose their initial analgesic effect within 1–2 years of permanent implantation. The author describes how psychological factors play an important role in the understanding of these findings. The results of this data analysis are in line with Doleys’s findings and illustrate the importance of psychological factors in the evolution of treatment outcomes.

Our results indicated that some of the previous findings are more difficult to reconcile. Feeling recovered and satisfied (as measured by the GPE) does not always correspond to an improvement in disability or general physical and psychological dysfunction. Evans et al. (2014) studied the variables that influence the GPE in patients with chronic neck pain. The authors stated that many patients believe that it is impossible to recover completely because of the perceived intractability of their physical condition. This has significant implications for long-term disability and health-seeking behaviors. The authors proposed a GPE model that captured several distinct domains relevant to patients with neck pain, which may be influenced by different factors. Feeling better compromises several aspects. Besides the resolution of symptoms, the redefinition of self and adjustment to pain as a part of life could reflect improvement in dealing with pain conditions.

### 4.3 Limitations

This study has several limitations. First, the patients may have provided socially desirable answers to complete the trial period and obtain a permanent implant. This may have biased the results and conclusions. Second, completing the chronic follow-up questionnaires was not mandatory but highly recommended. The compliance decreased over time and conclusions regarding the long-term functioning of implants are less reliable. Third, conclusions about the SCL-90-R for the whole Belgian population were not straightforward because the questionnaire was provided in three national languages. The SCL-90-R has different scales with different constitutions, validations, and norms for the Dutch-, French-, and German-speaking populations. This complicated the data analysis and the creation of uniform conclusions about the global Belgian population. Fourth, we lacked baseline pretrial information on the vital parameters of pain, sleep, and activity. Patients were invited to complete a diary on their pain, activity, and sleep starting from the first day of the trial period. This lack of data before the start of the trial period should be corrected in future procedure updates. In contrast, we have pre-evaluative psychological information; therefore, we can compare patients’ functioning before and after the trial, but we miss baseline information to draw conclusions about daily functioning (ADL) before and during the trial period. The Neuro-Pain^®^ platform invites patients to fill in a diary daily during the trial. Fifth, introducing an interactive register requires basic computer skills. Although the platform is intuitive and easy to use, some patients may require assistance to complete the questionnaires; diaries could be completed on paper and uploaded to the platform by the end of the trial period. Finally, the fact that most patients suffer from failed back surgery syndromes (PSPS type 2) and everyone is required to complete a 21 days trial period can also be seen by some as a limitation of this analysis. After all, this could introduce some possible bias in the analysis of the outcome parameters and predispose to the occurrence of infections during the prolonged trial period.

### 4.4 Future opportunities for the centralized interactive register

This first data analysis and report highlight several opportunities for further fine-tuning and improving the design, implementation, and use of the Neuro-Pain^®^ platform. First, a chronic follow-up module can be further developed to promote compliance. Second, the literature shows that functionality is a highly individualized concept that cannot be measured in a straightforward manner. Additional questionnaires or information about patients’ social contexts can help fill in these gaps. Third, changes should be made to the trial period structure to further decrease the risk of infection. Finally, data-driven feedback can help to refine the screening procedure. Regularly analyzing and evaluating the outcome measures and preimplantation psychosocial variables helped us critically review our screening and follow-up tools. Data cleaning can refine future analyses and reports of results.

## 5 Conclusion

First, this article provides an overview of how a web-based platform can be introduced to screen, evaluate, and follow-up patients during their treatment with SCS using a multidisciplinary approach. The Neuro-Pain^®^ project is an example of how the collaboration among medical specialists, psychologists, patients, and health insurers is facilitated through an innovative platform. This first data analysis and report highlight several opportunities for further fine-tuning and improving the design, implementation, and use of the Neuro-Pain^®^ platform.

Second, this article discusses the first outcome results of a 3 weeks trial period and the long-term follow-up of treatment with SCS based on a real-world dataset including 7,304 records off 6,170 patients. Although most of the results are based on a smaller Sub dataset (2,601 records off 2,572 patients), the results present some distinct conclusions. During the 3 weeks trial period, there were improvements in pain, activity level, and sleep quality. The intake of pain medication decreased during the trial period, and the general level of physical and psychological dysfunction in patients improved. Patients reported that their complaints had greatly improved, and they felt very satisfied with the results of the trial period. The results of the chronic follow-up questionnaire were inconsistent. During the first 6 months after the permanent implant, physical and psychological dysfunction, and pain-related disability increased, conversely, pain coping strategies evolved in a constructive way. Patients still felt relatively recovered and satisfied with their treatment; however, we observed a decline to a certain extent.

Finally, the relationship between psychological factors, “yellow flags,” and outcome measures was discussed. The following hypotheses were investigated: “Yellow flags are predictive of less recovery and less satisfaction after trial,” yellow flags are predictive of lower functioning after 6 months and 1.5 years.” Pain coping strategies and illness attitudes are predictive how recovered and satisfied patients feel with the results of the trial period. Feeling recovered and satisfied appear to be two different matters. Pain coping strategies, illness attitudes, general physical and psychological dysfunctioning, and the pain medication intake at the onset of the trial period (or “yellow flags”) are predictive of how disabled a patient feels after 6 months and 1.5 years after permanent implant.

## Data availability statement

The raw data supporting the conclusions of this article will be made available by the authors, without undue reservation.

## Ethics statement

The studies involving humans were approved by Ethical Committee of the Antwerp University Hospital (UZA) and also approved by the National Belgian E-Health Privacy Committee (regarding the structure, data input and data transmission of all medical information contained in the register). The studies were conducted in accordance with the local legislation and institutional requirements. The participants provided their written informed consent to participate in this study.

## Author contributions

LB: Methodology, Supervision, Writing – original draft, Writing – review & editing, Data curation. ER: Data curation, Methodology, Writing – original draft, Formal analysis, Software, Validation, Visualization. FL: Methodology, Conceptualization, Investigation, Writing – review & editing. MMo: Writing – review & editing. J-PB: Writing – review & editing. BBi: Writing – review & editing. BBr: Writing – review & editing. MP: Writing – review & editing. TTu: Writing – review & editing. MMa: Writing – review & editing. TTh: Writing – review & editing. JZ: Writing – review & editing. AB: Writing – review & editing. EC: Writing – review & editing. OC: Writing – review & editing. JV: Writing – review & editing. HL: Writing – review & editing. ML: Writing – review & editing. GH: Writing – review & editing, Conceptualization, Funding acquisition, Investigation, Methodology, Project administration, Resources, Software, Supervision, Validation, Visualization, Writing – original draft.
